# Photosynthetic hydrogen production by droplet-based microbial micro-reactors under aerobic conditions

**DOI:** 10.1038/s41467-020-19823-5

**Published:** 2020-11-25

**Authors:** Zhijun Xu, Shengliang Wang, Chunyu Zhao, Shangsong Li, Xiaoman Liu, Lei Wang, Mei Li, Xin Huang, Stephen Mann

**Affiliations:** 1 MIIT Key Laboratory of Critical Materials Technology for New Energy Conversion and Storage, School of Chemistry and Chemical Engineering, Harbin Institute of Technology, Harbin, 150001 China; 2 Max Planck Bristol Centre for Minimal Biology, Centre for Protolife Research and Centre for Organized Matter Chemistry, School of Chemistry, University of Bristol, Bristol, BS8 1TS UK

**Keywords:** Metabolic engineering, Photosynthesis, Hydrogen energy, Self-assembly

## Abstract

The spontaneous self-assembly of multicellular ensembles into living materials with synergistic structure and function remains a considerable challenge in biotechnology and synthetic biology. Here, we exploit the aqueous two-phase separation of dextran-in-PEG emulsion micro-droplets for the capture, spatial organization and immobilization of algal cells or algal/bacterial cell communities to produce discrete multicellular spheroids capable of both aerobic (oxygen producing) and hypoxic (hydrogen producing) photosynthesis in daylight under air. We show that localized oxygen depletion results in hydrogen production from the core of the algal microscale reactor, and demonstrate that enhanced levels of hydrogen evolution can be achieved synergistically by spontaneously enclosing the photosynthetic cells within a shell of bacterial cells undergoing aerobic respiration. Our results highlight a promising droplet-based environmentally benign approach to dispersible photosynthetic microbial micro-reactors comprising segregated cellular micro-niches with dual functionality, and provide a step towards photobiological hydrogen production under aerobic conditions.

## Abstract (web summary)

The development of techniques capable of orchestrating the assembly of living cells into multicellular ensembles with synergistic and function is challenge. Here, the authors construct algal or algal/bacterial cells-based core shell-like structure based on aqueous two-phase system for synergic photosynthetic H_2_ production.

## Introduction

The development of new techniques capable of orchestrating the assembly of living cells into multicellular ensembles is a fundamental requirement for applications in tissue engineering and cell therapy^1–4^. Alongside micro-engineering procedures such as microfluidics, chemically mediated droplet approaches are currently being developed for the spontaneous organization of living cells or their artificial cell-like counterparts into tissues and prototissue assemblages, respectively. In particular, water-in-oil emulsion droplets have been exploited for the programmed assembly of extended networks of synthetic protocells^5–8^, and aqueous two-phase systems comprising demixed polymers or complex coacervates^9,10^ used to capture subcellular organelles^11,12^, bacteria^13^, viruses^4^, and mammalian cells^14,15^. In the latter approach, the inherent biocompatibility of many water-in-water (w/w) emulsion systems and absence of organic solvents and crosslinking agents offers a promising way to the design and construction of multicellular aggregates^16^ for applications in biomedicine^17,18^, the printing of tumour spheroids^19^, assembly of heterogeneous stem cell niches^20^, and development of artificial models of cellular assembly^21–23^. In contrast, the use of aqueous two-phase separated droplets as environmentally benign microscale platforms for the spontaneous assembly and spatial segregation of water-dispersible microbial micro-reactors has received minimal attention.

**Fig. 1:**
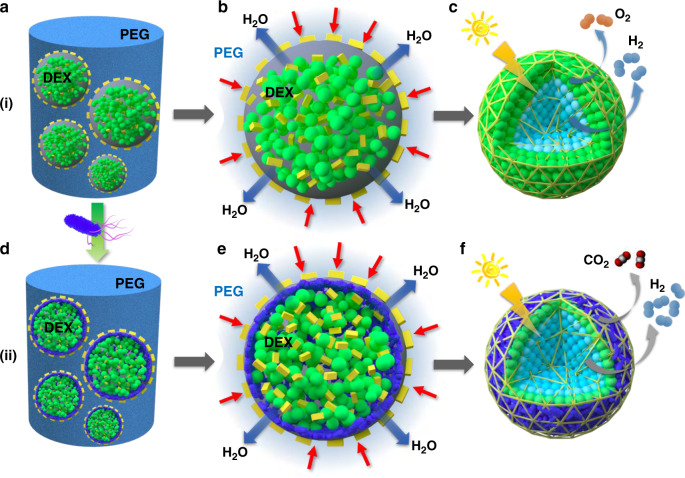
Schematic illustration showing the assembly, spatial organization and dual functionality of multicellular droplet-based living micro-reactors. (i) Algal cell-based spheroids: **a** Large numbers of *Chlorella* algal cells (green spheres) are spontaneously captured within w/w dextran-in-PEG micro-droplets by emulsification in the presence of denatured BSA micro-particles (yellow rectangles). **b** Cell-containing emulsion droplets are hyperosmotically compressed (red arrows) by transfer to a concentrated PEG solution to produce robust multicellular spheroids comprising a closely packed aggregate of algal cells immobilized in a dextran/BSA hydrogel matrix. **c** Cell-mediated depletion of oxygen over time in the hydrogel matrix (yellow triangular network) generates hypoxic (interior, cyan) and aerobic (surface, green) micro-niches due to light shading of the algal cells in the core domain by the outer shell of *Chlorella* cells. Depending on the size of the spheroids, photosynthetic oxygen generation is decreased in the core such that respiration becomes dominant over photosynthesis resulting in the net depletion of cellular storage compounds, hypoxic conditions, hydrogenase activity and hydrogen production in daylight under air. Corresponding reactions: Shell domain: H_2_O → ½O_2_ + 2H^+^ + 2e^−^ (PSII). Core domain: H_2_O → ½O_2_ + 2H^+^ + 2e^−^ (PSII) and 2H^+^ + 2e → H_2_ (hydrogenase). (ii) Algal/bacterial hybrid spheroids: (**d**) Preparation of the droplets using mixtures of *Chlorella* and PEGylated *E. coli* cells (blue rods) results in a spatially segregated arrangement of photosynthetic algal cells enclosed by a thin oxygen-depleting layer of respiratory bacterial cells. **e** Hyperosmotic compression results in consolidation and immobilization of the two cellular micro-niches. **f** In daylight under air, the binary community acts synergistically to enhance the levels of hydrogen production produced by hypoxic photosynthesis in the core of the multicellular hybrid micro-reactor. Corresponding reactions: Shell domain: H_2_O → ½O_2_ + 2H^+^ + 2e^−^ (PSII) and O_2_ → CO_2_ (respiration from *E. coli*). Core domain: H_2_O → ½O_2_ + 2H^+^ + 2e^−^ (PSII) and 2H^+^ + 2e^−^ → H_2_ (hydrogenase).

Given that the renewable and biological production of hydrogen from solar energy is attracting considerable interest, in this study, we exploit the aqueous two-phase separation of dextran-in PEG emulsion droplets for the capture, spatial organization and immobilization of living algal cells to produce dispersible microscale microbial reactors capable of both aerobic and hypoxic photosynthesis in daylight under air (Fig. 1a–c). The capture of large numbers of cells in each w/w emulsion droplet occurs spontaneously and at high efficiency to generate a suspension of droplet-based algal colonies that are subsequently hyperosmotically compressed into closely packed multicellular spheroids. We show that the dual photosynthetic behaviour originates from the onset of a hypoxic micro-niche within the interior of the spheroids such that the production of oxygen and hydrogen (via hydrogenases^24–27^) occurs respectively at the surface or within the core of the dispersed bioreactors. We develop the methodology to fabricate microbial micro-reactors comprising spatially segregated communities of algal and non-photosynthetic bacterial cells that operate synergistically through the interplay of hypoxic photosynthesis and aerobic respiration to enhance the level of hydrogen production under air (Fig. 1d–f). Taken together, our methodology offers a proof-of-principle for utilizing aqueous two-phase separated droplets as vectors for controlling algal cell organization and photosynthesis in synthetic micro-spaces and provides a step towards the bottom-up assembly of photobiological micro-reactors with multiple functionalities.

## Results

### Construction of algal multicellular spheroids

**Fig. 2:**
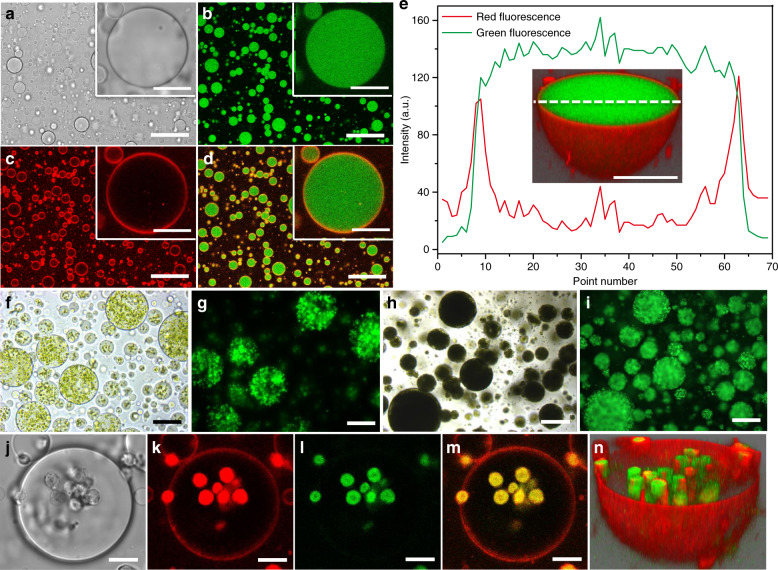
Capture of *Chlorella* cells within w/w emulsion droplets. **a** Confocal bright-field images of a population of dextran-in-PEG micro-droplets and a single droplet (inset) stabilized by denatured BSA micro-particles. **b**, **c** Corresponding confocal fluorescence images of droplets displayed in (**a**) showing presence of FITC-dextran throughout the droplets (**b**, green fluorescence) and a thin shell of Nile red-stained BSA particles at the droplet surface (**c**, red fluorescence). (**d**) Overlay channel of (**b**, **c**). Scale bars in (**a**–**d**), 100 and 10 μm (insets). **e** Plot of red and green fluorescence intensities across a single droplet. Inset shows the corresponding 3D confocal scanning image; staining as in (**d**); scale bar, 10 μm (inset). **f**–**i** Optical microscopy images (**f**, **h**) and corresponding green fluorescence microscopy images (**g**, **i**) of w/w emulsion droplets prepared in the presence of low (**f**, **g**; 1.2 × 10^8^ cells/mL) and high (**h**, **i**; 3.3 × 10^9^ cells/mL) *Chlorella* cell densities. The algal cells were treated with fluorescein diacetate (FDA); viable cells gave rise to the hydrolytic release of fluorescein (green fluorescence). Scale bars in (**f**, **g**) and (**h**, **i**), 100 and 200 μm, respectively. **j**–**n** Confocal bright-field microscopy image (**j**) and corresponding red and green confocal fluorescence microscopy images (**k**, **l**), red/green overlay (**m**) and 3D image (**n**) of a single droplet showing discrete *Chlorella* cells captured within the dextran-rich interior. Samples were prepared at a low cell number density (6.9 × 10^7^ cells/mL) using non-labelled dextran and Nile Red-stained BSA particles. The captured algal cells display red and green fluorescence due to intracellular chlorophyll and cell-mediated release of fluorescein, respectively. The Nile Red-stained BSA particles are observed as a red fluorescence ring at the w/w droplet interface. Scale bars in (**j**–**m**), 10 μm. All relevant experiments were performed independently at least three times with similar results. Source data underlying Fig. 2e are provided as a Source Data file.

Aqueous solutions of dextran (Dex, M_W_ 500 kDa) and polyethylene glycol (PEG, M_W_ 20 kDa) were sheared at room temperature at a volume ratio of 1:9 to produce a w/w emulsion comprising dextran-rich micro-droplets dispersed in a PEG-rich continuous phase. Droplet coalescence was minimized by the addition of specifically synthesized microparticles of denatured BSA (Supplementary Figs. 1–3) to the dextran phase prior to emulsification (Supplementary Fig. 4). As a consequence, stable droplets with mean diameters ranging between ~20 and 180 μm could be prepared under different shearing conditions (Supplementary Fig. 5). Location of the BSA particles at the droplet surface was confirmed by labelling the denatured protein particles with Nile Red and tagging the dextran with fluorescein isothiocyanate (FITC-Dex), and then imaging the emulsion droplets by confocal fluorescence microscopy (Fig. 2a–e).

The dextran-in-PEG micro-droplets were used as a dispersed platform to capture high loadings of the photosynthetic alga *Chlorella pyrenoidosa*. This was achieved by mixing the denatured BSA microparticle stabilizer with a solution of dextran containing high numbers of the *Chlorella* cells (Supplementary Fig. 6), followed by injection of the mixture into a rapidly stirred PEG solution to produce a w/w emulsion (Supplementary Fig. 7). In contrast, no stable cell-loaded emulsion droplets were produced when PEG was used as the loading phase (Supplementary Fig. 8). The presence of the algal cells had minimal effect on polymer demixing or droplet stabilization such that populations of stable dextran-enriched droplets containing adjustable mean cell number densities were produced (Fig. 2f–i). Solubilization of the algal cells within the emulsion droplets was attributed to the increased hydrophilicity of dextran compared with PEG. Significantly, when the trapped algal cells were exposed to fluorescein diacetate (FDA), a high green fluorescence intensity was observed throughout the droplets confirming that the *Chlorella* cells were alive after capture (Fig. 2g, i). This was consistent with FDA-stained images of discrete individual cells captured at low number densities within the droplets that displayed superimposed regions of red and green fluorescence due to intracellular chlorophyll and cell-mediated release of fluorescein, respectively (Fig. 2j–n).

Under the conditions employed, almost all the algal cells were encapsulated in the w/w emulsion droplets with minimal numbers of free *Chlorella* cells observed in the PEG-rich continuous phase. For example, preparing samples using high concentrations of algal cells (3.3 × 10^9^ cells/mL) at a stirring rate of 100 rpm produced droplets with mean diameters of *ca*. 170 μm that typically contained ~9000 closely packed *Chlorella* cells per droplet. Increasing the stirring rate used for emulsification up to 400 rpm progressively decreased the mean size of the droplets without affecting the entrapment of the algal cells (Supplementary Fig. 9).

**Fig. 3:**
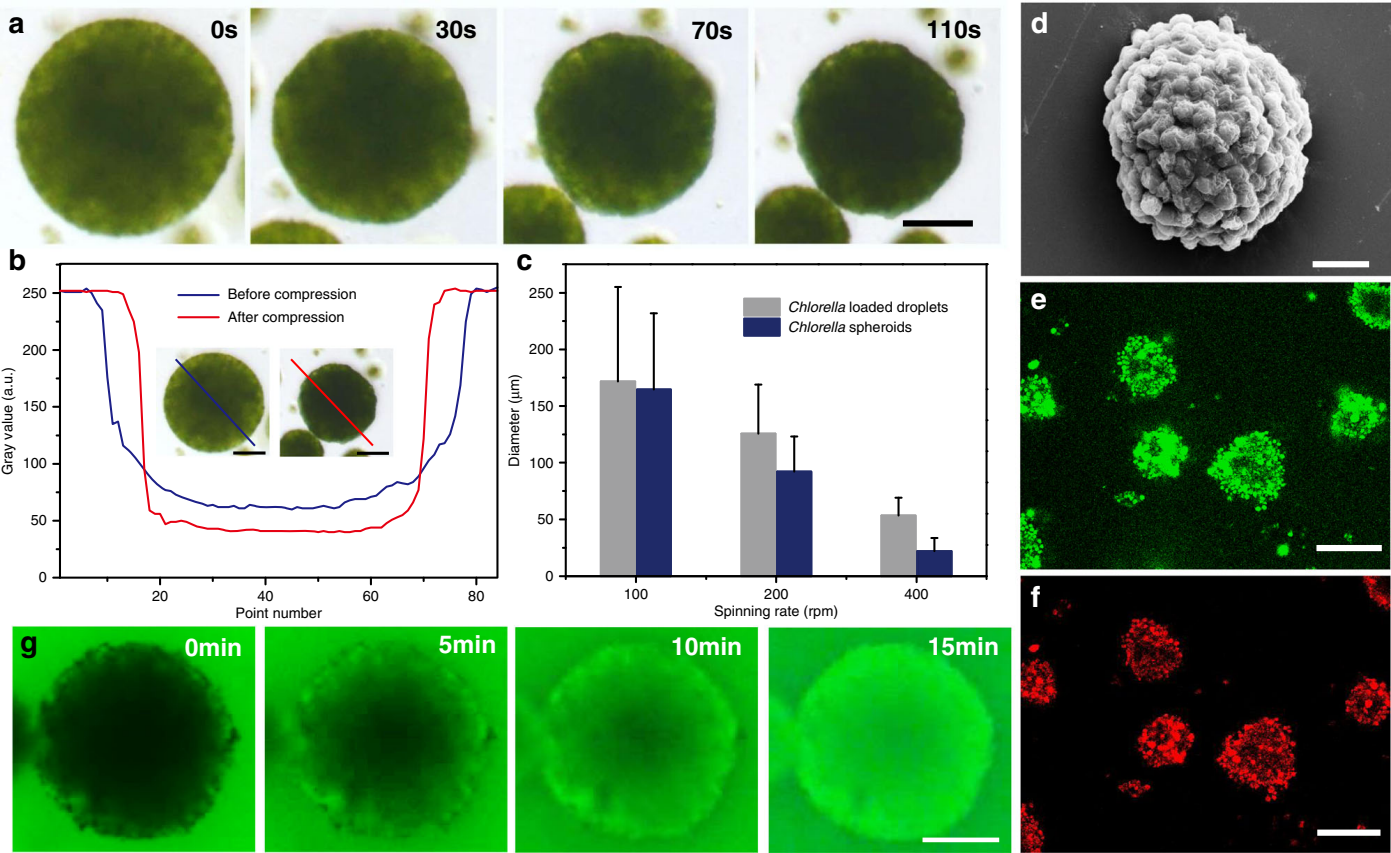
Formation of *Chlorella* cell-based spheroids. **a** Time sequence of optical microscopy images of a single dextran-in-PEG micro-droplet containing entrapped *Chlorella* cells and undergoing shrinkage after immersion in hyperosmotic PEG solution at 0, 30, 70 and 110 s; scale bar, 50 μm. **b** Corresponding plots of brightness line profiles before (blue; *t* = 0 s) and after (red; *t* = 110 s) hyperosmotic compression. Scale bar, 50 μm (inset). **c** Plots of diameters of *Chlorella*-loaded droplets prepared at different spinning rates, before (navy blue) and after (deep cyan) hyperosmotic compression. Data are presented as mean values ± SD, error bars indicate standard deviations. **d** SEM image of a single multicellular spheroid; scale bar, 10 μm. **e**, **f** Confocal microscopy fluorescence images of *Chlorella* cell-based spheroids after FDA staining showing viable algal cells after hyperosmotic compression within the spheroids (**e**); red fluorescence is from intracellular chlorophyll (**f**); scale bars, 75 μm. **g** Sequence of fluorescence microscopy images showing slow diffusion of fluorescein into a single *Chlorella* cell-based spheroid recorded at *t* = 0, 5, 10 and 15 min after addition of the dye; scale bar, 30 μm. All relevant experiments were performed independently at least three times with similar results. Source data underlying Fig. 3b, c are provided as a Source Data file.

Given the high entrapment efficiency, we sought to transform the individual *Chlorella* cell-containing dextran-in-PEG droplets into robust multicellular spheroids as a step towards the fabrication of a living microbial micro-reactor. We achieved this by transferring the algal cell-loaded w/w emulsion droplets into a hyperosmotic PEG solution (50%, w/w, M_W_ 2000 Da) to shrink the individual droplets (Fig. 3a–c and Supplementary Fig. 10). Typically, droplets with mean sizes of 171, 126 and 53 μm were hyperosmotically compressed to respective values of 165, 92 and 22 μm typically within 110 s (Fig. 3c and Supplementary Fig. 11). As a consequence, the entrapped *Chlorella* cells were compacted into discrete robust spheroids consisting of closely packed 3D assemblies (Fig. 3d). The spheroids were negatively charged (Supplementary Fig. 12) and stable when transferred into water (Supplementary Fig. 13). In contrast, dilution of the droplets before hyperosmotic compression resulted in disruption of the loosely packed clusters of algal cells (Supplementary Fig. 14). The presence of large numbers of proliferating living algal cells in the spheroids was confirmed by FDA staining (Fig. 3e, f) and the slow but progressive increase in the intracellular chlorophyll concentration (Supplementary Fig. 15).

Irreversible formation of the microbial spheroids was attributed principally to the embedding of the closely packed multicellular clusters in a denatured BSA hydrogel produced by the local concentration of the microparticle emulsion stabilizer during osmotic shrinkage of the dextran-in-PEG droplets (Supplementary Figs. 16 and 17). This was confirmed by determining the spheroid stability for samples produced from *Chlorella* cell-loaded droplets that were prepared by adding the stabilizer protein microparticles to the PEG phase instead of dextran prior to emulsification. In this case, most of the denatured BSA particles remained in the continuous PEG phase (Supplementary Fig. 18) such that only loosely packed aggregates of *Chlorella* cells were obtained in the dextran-enriched spheroids produced after hyperosmotic compression (Supplementary Fig. 19). Immobilization of the closely packed *Chlorella* cells within the denatured BSA hydrogel/dextran matrix impeded the diffusion of small molecules into the spheroid interior; for example, it took *ca*. 15 min for fluorescein to completely access all regions of the spheroids with a mean size of 92 μm (Fig. 3g and Supplementary Fig. 20).

### Hypoxic and aerobic photosynthesis in algal cell micro-reactors under air

**Fig. 4:**
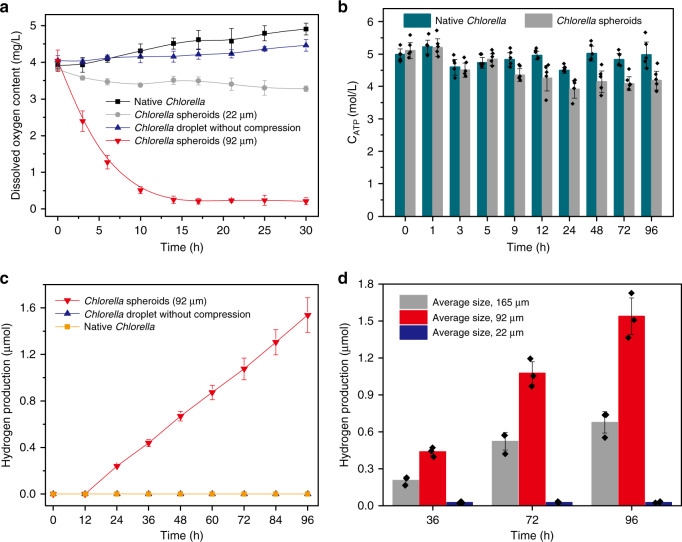
Hypoxic and aerobic photosynthesis in algal cell micro-reactors under air. **a** Time-dependent measurements of dissolved oxygen concentration in suspensions of native *Chlorella* cells (black squares), *Chlorella*-loaded w/w dextran-in-PEG emulsion droplets (blue triangles), and *Chlorella* multicellular spheroids with a mean size of 22 μm (grey spheres) or 92 μm (red inverted triangles). All samples were in sealed vials and exposed to daylight at an intensity of 100 μE m^−2^ s^−1^. Data are presented as mean values ± SD, error bars indicate standard deviations (*n* = 3). **b** Time-dependent changes in ATP concentration of native free *Chlorella* cells and *Chlorella* cells within closely packed spheroids (mean size, 92 μm). Data are presented as mean values ± SD, error bars indicate standard deviations (*n* = 5). **c** Time-dependent measurements of hydrogen concentration in suspensions of native *Chlorella* cells (orange squares), *Chlorella*-loaded w/w dextran-in-PEG emulsion droplets (blue triangles (superimposed on orange squares)), and *Chlorella* multicellular spheroids with a mean size of 92 μm (red inverted triangles). All samples were in sealed vials and exposed to daylight at an intensity of 100 μE m^−2^ s^−1^. Data are presented as mean values ± SD, error bars indicate standard deviations (*n* = 3). **d** Time-dependent production of hydrogen for *Chlorella* multicellular spheroids prepared with mean sizes of 22 (blue), 92 (red) or 165 μm (black). Data are presented as mean values ± SD, error bars indicate standard deviations (*n* = 3). Source data are provided as a Source Data file.

Given the presence of living algal cells within the spheroids, we sought to exploit the microbial constructs as multicellular micro-reactors capable of light-induced bioactivity. We first exposed native *Chlorella* cells or emulsion droplets containing loosely packed aggregates of *Chlorella* cells to daylight at an intensity of 100 μE m^−2^ s^−1^ and monitored the oxygen content over time in sealed vials. In both cases, the algal cells were photosynthetically active with oxygen levels increasing over a period of 30 h (Fig. 4a). Similarly, negligible changes in dissolved oxygen content were observed for closely packed algal cells in hyperosmotically compressed spheroids with relatively small sizes (22 μm) (Fig. 4a), indicating that embedding of the *Chlorella* cells within the BSA/dextran matrix of the emulsion droplets did not significantly curtail their photosynthetic activity. However, increasing the mean size of the light-exposed spheroids from 22 to 92 μm resulted in a decrease in the dissolved oxygen concentration from 4.04 to 0.25 mg/L over *ca*. 12 h (Fig. 4a) and time-dependent decreases of the intracellular ATP concentrations (Fig. 4b). This suggested that as the spheroid core became larger, respiration became dominant over photosynthesis such that there was a net consumption of oxygen molecules. We attributed this transformation to the onset of a hypoxic micro-environment within the centre of the spheroids due to partial shielding of the buried algal cells to the light source as well as restrictions in the diffusion of atmospheric oxygen into the core regions. As a consequence, *Chlorella* cells within each micro-reactor became segregated into hypoxic or aerobic micro-niches depending on whether they were located in the core or shell of the spheroid, respectively.

Given that hydrogenase activity is induced in *Chlorella* cells under hypoxic photosynthetic conditions^26,27^, we investigated whether the algal cells confined within the core of the spheroids could also utilize photosynthetic electrons for hydrogen production in air to produce microbial micro-reactors with dual functionality. As *Chlorella* cells located in the surface and near-surface regions of the spheroids retained their photosynthetic oxygen productivity, we used larger spheroids (mean size, 92 μm) to maximise the levels of evolved hydrogen from the micro-niche in the core of the multicellular micro-reactors (Fig. 1c). Time-dependent measurements showed a linear increase in the hydrogen concentration over a period of 96 h for spheroids exposed to daylight with an intensity of 100 μE m^−2^ s^−1^ (Fig. 4c). Production of hydrogen occurred after an initial induction period of 12 h, which we attributed to the time required for the onset of hypoxic conditions in the core of the spheroids.

In contrast, minimal levels of hydrogen production were observed for native *Chlorella* cells, or when non-osmotically compressed dextran-in-PEG emulsion droplets containing loosely aggregated algal cells were exposed to light in air (Fig. 4c). Similarly, the amount of hydrogen produced in air was negligible when the mean size of the *Chlorella* cell-based spheroids was decreased from 92 to 22 μm (Fig. 4d), in agreement with the relatively high oxygen production under these conditions due to the increased percentage of algal cells in the aerobic surface regions (Fig. 4a). Interestingly, the amount of hydrogen produced in different time periods was reduced by *ca*. 50% when the mean size of the microbial spheroids was increased from 92 to 165 μm (Fig. 4d). We attributed this to an increased level of shielding of the light from the algal cells located deep within the core of the spheroids such that hypoxic photosynthesis was increasingly curtailed.

### Synergistic hydrogen production in *Chlorella*/*E. coli* hybrid micro-reactors

The above studies indicated that the droplet-mediated organization of living algal cells into closely packed discrete multicellular clusters generated changes in local oxygen levels that gave rise to core and shell segregated micro-niches of hypoxic and aerobic photosynthesis, respectively. The corresponding levels of hydrogen or oxygen production in air were determined by the cell number distributions in the core and surface regions, respectively, which given functional levels of light penetration were dependent on the spheroid size, leading to an optimum micro-reactor size for hydrogen production under air. However, even under optimal conditions, the hydrogen production rate for the algal cell spheroids in air was approximately three times lower than the rate determined under argon (0.25 and 0.86 μmol H_2_ (mg chlorophyll)^−1^ h^−1^, respectively) (Supplementary Fig. 21), indicating that around two-thirds of the *Chlorella* cells were not contributing to hydrogen evolution under an aerobic environment. As these cells occupied the aerobic surface and near-surface regions of the spheroids, we sought to establish an oxygen depletion zone around the shell domain to increase the percentage of *Chlorella* cells engaged in hypoxic photosynthesis to a level that provided more effective rates of hydrogen production in air.

**Fig. 5:**
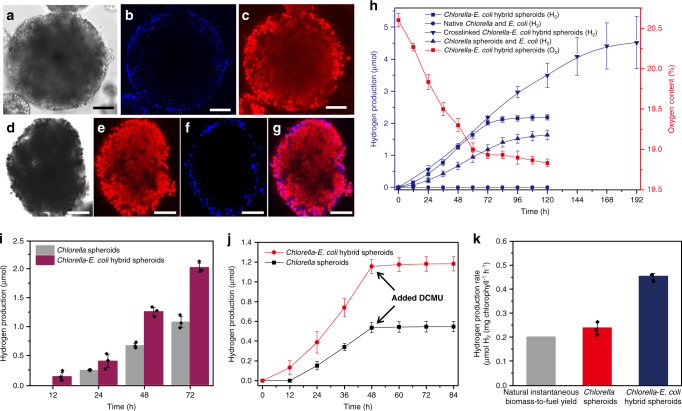
Synergistic hydrogen production in *Chlorella/E. coli* hybrid micro-reactors in air. **a**–**c** Bright-field image (**a**), and blue and red confocal fluorescence images (**b**, **c**) of a single w/w dextran-in-PEG emulsion droplet with captured red algal and surface-adsorbed bacterial cells. PEGylated *E. coli* cells are labelled with Atto425 (blue fluorescence); red fluorescence is from chlorophyll present within the *Chlorella* cells; scale bars, 25 μm. Samples were prepared at 200 rpm; mean droplet size = 116 μm. **d**–**f** Confocal bright field image of a single *Chlorella*/*E. coli* multicellular spheroid (**d**) and corresponding red (**e**) and blue (**f**) confocal fluorescence images. PEGylated *E. coli* cells are labelled with Atto425 (blue fluorescence) and are located specifically at the spheroid surface while the *Chlorella* cells (red fluorescence, chlorophyll) are closely packed throughout the hybrid spheroid. **g** Overlay image of (**e**) and (**f**) showing the core-shell spatial organization. Scale bars in (**d**–**g**), 20 μm. Samples were prepared at 200 rpm; mean spheroid size = 90.8 μm. **h** Time-dependent measurements of hydrogen concentration in mixed suspensions of native *Chlorella* and *E. coli* cells (blue circles), *Chlorella* spheroids and free *E. coli* cells (blue triangles), *Chlorella*/*E. coli* multicellular spheroids (blue squares) and Dex-CHO crosslinked *Chlorella*/*E. coli* multicellular spheroids (blue inverted triangles); corresponding decrease in oxygen content for the algal/bacterial cell spheroids is also shown (red squares). All samples were in sealed vials and exposed to daylight at an intensity of 100 μE m^−2^ s^−1^. Data are presented as mean values ± SD, error bars indicate standard deviations (*n* = 3). **i** Time-dependent plots of hydrogen production for *Chlorella* cell-based spheroids (grey columns) and *Chlorella/E. coli* hybrid core-shell spheroids (purple columns) at different time periods. Data are presented as mean values ± SD, error bars indicate standard deviations (*n* = 3). **j** Time-dependent plots of hydrogen production for *Chlorella* spheroids (black) and *Chlorella/E. coli* hybrid core-shell spheroids (red). Data are presented as mean values ± SD, error bars indicate standard deviations (*n* = 3). **k** Plots of average hydrogen production rate for *Chlorella* multicellular spheroids (red) and *Chlorella*/*E. coli* hybrid spheroids (blue) and for the natural instantaneous biomass-to-fuel yield (grey). Production rates for the spheroids were determined between time points of 24 and 72 h. Data are presented as mean values ± SD, error bars indicate standard deviations (*n* = 3). All relevant experiments were performed independently at least three times with similar results. Source data underlying Fig. 5h–k are provided as a Source Data file.

To integrate an efficient oxygen depletion layer at the surface of the photosynthetically active spheroids, we developed a procedure for the spontaneous core-shell spatial organization of a community of photosynthetic (*Chlorella*) and non-photosynthetic (*Escherichia coli*) cells within each microbial micro-reactor (Fig. 1d–f). We reasoned that spheroids comprising an inner core of algal cells and outer shell of respiring bacterial cells would exhibit enhanced rates of hypoxic photosynthesis due to the continuous respiratory uptake of oxygen within the bacterially enriched surface regions of the multicellular hybrid micro-reactor. Moreover, as excreted algal-derived organic matter can be used as a substrate for bacterial respiration in natural and co-cultured algal/bacterial communities^28,29^, we speculated that nutrient exchange between the co-located cells would lead to an enhancement in photobiological production. To achieve the spontaneous spatial segregation of the *E. coli* cells specifically around the *Chlorella* cells during capture of the cell community by the w/w dextran-in-PEG droplets, we PEGylated the external wall of the bacterial cells prior to emulsification at 200 rpm to increase their solubility in the PEG continuous phase (Supplementary Fig. 22). As a consequence, the *E. coli* cells were adsorbed predominantly at the dextran/PEG interface and not sequestered into the interior of the dextran-enriched emulsion droplets, while the *Chlorella* cells were captured at high concentrations (Fig. 5a–c and Supplementary Fig. 23). Subsequent hyperosmotic shrinkage of the hybrid droplets produced multicellular spheroids (mean size = 90.8 μm) with an algal/bacterial core-shell arrangement in which the closely packed *Chlorella* cells were encased in a thin layer of *E. coli* cells (Fig. 5d–g). In contrast, spheroids prepared from mixtures of algal and non-PEGylated bacterial cells exhibited a homogeneous distribution of the binary cell community (Supplementary Fig. 24).

Exposure of the hybrid spheroids to daylight in air at an intensity of 100 μE m^−2^ s^−1^ resulted in the rapid onset of hydrogen evolution that increased progressively in the sealed vial to reach a steady-state value after 84 h (Fig. 5h). Simultaneously, oxygen levels in the vial decreased from 20.6 to 18.9% over 60 h (Fig. 5h), consistent with the onset of hypoxic photosynthesis in the closely packed *Chlorella* cells. In contrast, mixtures of the *Chlorella* spheroids along with free *E. coli* cells showed no enhanced photosynthetic hydrogen production when compared with yields observed for aqueous suspensions of *Chlorella* spheroids alone (Figs. 5h and 4c). The hybrid spheroids exhibited a reduced induction period and increased initial level of hydrogen production (0–12 h, 2.02 μmol, respectively) compared with micro-reactors containing only *Chlorella* cells (12–24 h, 1.08 μmol) (Fig. 5i). In both cases, the addition of 3-(3,4-dichlorophenyl)-1,1-dimethylurea (DCMU) inhibited hydrogen production (Fig. 5j). This was consistent with the simultaneous DCMU-mediated disruption of the PSII pathway and inhibition of respiration, and implied that both electron transfer and oxygen-scavenging were important in determining hydrogen production. Overall, the mean rate of hydrogen production in the hybrid spheroids was 2.2 times higher than determined for the instantaneous biomass-to-fuel yield observed in Nature (0.20 μmol H_2_ (mg chlorophyll)^−1^ h^−1^) (Fig. 5k)^30^.

Taken together, the above results were consistent with an enhanced level of hypoxic photosynthesis in the hybrid spheroids due to the respiratory uptake of oxygen by the surface *E. coli* cells and formation of a distinct oxygen-depleted micro-niche of *Chlorella* cells in the micro-reactor interior. As the *Chlorella* and *Chlorella*/*E. coli* micro-reactors were stabilized only by non-covalent interactions along with in situ compaction and hydrogelation of the denatured BSA microparticles during hyperosmotic compression, the spheroids disassembled over time as the cell colonies proliferated (Supplementary Figs. 25–27). As a consequence, hydrogen production was terminated typically within 72 h even though levels of cell viability after disassembly at 108 h were 68% and 96% for *E. coli* and *Chlorella* cells, respectively (Supplementary Fig. 28). To prolong the lifetime of the hybrid bioreactor, we included an aldehyde-functionalized dextran (Dex-CHO) into the w/w dextran-rich emulsion droplets to chemically crosslink the BSA protein hydrogel in situ. The resulting spheroids were physically more robust and displayed extended periods of hydrogen production over 168 h (Fig. 5h and Supplementary Figs. 29, 30).

## Discussion

In conclusion, we developed a facile strategy for the preparation of populations of discrete microscale microbial reactors with spatially segregated multicellular micro-niches capable of aerobic or hypoxic photosynthesis at room temperature in air. Large numbers of *Chlorella* algal cells were spontaneously captured within BSA-stabilized w/w dextran-in-PEG emulsion droplets and then compacted into closely packed spheroidal aggregates by hyperosmotic shrinkage and in situ formation of a BSA/dextran hydrogel matrix. Depending on the size of the spheroids, cells within the core of the aggregates switched from aerobic to hypoxic photosynthesis as the oxygen content of the internal micro-environment became depleted and hydrogenase activity ensued. As a consequence, considerable levels of hydrogen were produced from the algal cell micro-reactors when exposed to daylight in air. Given that oxygen and hydrogen production were determined by the number of *Chlorella* cells distributed between the shell and core micro-niches, respectively, we increased the level of hypoxic photosynthesis in the micro-reactors by using the droplet methodology to spontaneously encase the closely packed algal cells within a continuous layer of non-photosynthetic bacteria. In this way, we coupled aerobic respiration and hypoxic photosynthesis synergistically to generate hydrogen from the spatially organized microbial community at rates that were higher than reported for the instantaneous biomass-to-fuel yields observed in Nature. Significantly, the *E. coli*-enriched outer shell not only acted as a shield of the light source but also consumed oxygen produced from algal cell photosynthesis in the core of the spheroids, both of which facilitated H_2_ biosynthesis provided that the cellular storage compounds were not depleted and that the spheroids did not disassemble due to continued cell proliferation. In the latter case, crosslinking of the hydrogel matrix within the multicellular spheroids resulted in an extension in continuous hydrogen production to 168 h.

Overall, our methodology provides a proof-of-principle for utilizing aqueous two-phase separated droplets as vectors for controlling algal cell organization and photosynthesis in synthetic micro-spaces. The procedure is facile and capable of high throughputs for modulating algal cell functionality towards hydrogen production without impairing the viability of the living cells. Moreover, it should be possible to combine our methodology with more complex bioengineering approaches involving sulfur deprivation^24^, genetically modified oxygen-tolerant [FeFe]-hydrogenases^25^ or cellular surface modifications^27^. Compared with synthetic hydrogen-producing systems^30^, the limited rates and yields in the multicellular spheroids remain challenging aspects of future work. In this regard, incorporating chemical-based hydrogen-generating machinery^31,32^ or antennae-reduced mutants^33^ into the algal cell spheroids could be promising strategies. More generally, our approach provides the possibility for modulating the functionality of other living cells; for example, the droplet-based microbial systems can be readily extended towards ethanol production via the programmed capture of large numbers of yeast cells within the multicellular spheroids (Supplementary Fig. 31).

Taken together, our results highlight a promising environmentally benign approach to dispersible living cell microbial micro-reactors and provide a step towards promoting photobiological green energy development under natural aerobic conditions. More broadly, the use of aqueous two-phase separated droplets to selectively sequester and spatially organize different types of cells within discrete microscale packages could provide opportunities in biomedical applications such as cell therapy and tissue engineering, provide a model system for controlling the directed 3D assembly of living cells, and serve as a general method for the construction of synthetic protocell colonies and prototissues.

## Methods

### Characterization

Optical and fluorescence microscopy were performed on a Leica DMI8 manual inverted fluorescence microscope at ×10, ×20 and ×40 magnification. Scanning electron microscopy (SEM) images were obtained on a SU8000 instrument with the samples sputter-coated with 10 nm platinum. Transmission electron microscopy (TEM) images were undertaken on a JEM-1400, using a filament at 120 kV in bright field mode. Fourier Transform infrared spectroscopy (FTIR) measurements were performed on PerkinElmer spectrometer with a LiTaO_3_ detector (Spectrum Two, USA). Confocal scanning laser microscopy (CSLM) images were obtained on a Leica SP8 confocal laser scanning microscope attached to a Leica DMI 6000 inverted epifluorescence microscope. The pH measurements were made with a Seven Compact meter (Mettler Toledo, Sui). Emulsification was undertaken using a Vortex instrument (IKA, Germany). Circular dichroism (CD) spectral measurements were conducted using a Chirascan Plus CD spectrometer (Applied Photophysics Ltd, Leatherhead, UK). Samples were diluted with DI water to concentrations of 0.6 mg/mL in quartz cuvettes of 1 mm pathlength. Fluorescence spectroscopy was performed by a fluorescence spectrophotometer (PerkinElmer, USA, LS 55). Samples were diluted to a protein concentration of 1 mg/mL, and recorded at an excitation wavelength of 295 nm and slit widths of 6.0 nm (Excitation slit) and 3.0 nm (Emission slit).

### Cell cultures

*Chlorella pyrenoidosa* cells were cultured in TAP medium containing 2 × 10^−2^ M Tris, 7 × 10^−3^ M NH_4_Cl, 8.3 × 10^−4^ M MgSO_4_·7H_2_O, 4.5 × 10^−4^ M CaCl_2_·7H_2_O, 1.65 × 10^−3^ M K_2_HPO_4_, 1.05 × 10^−3^ M KH_2_PO_4_, 1 mL/L Hunter’s trace elements and 1 mL/L glacial acetic acid. The medium pH was adjusted to 7.0. The cells were cultured in 0.5 L of TAP medium at 25 °C with alternative daytime (12 h, 40  μE m^−2^ s^−1^) and night (12 h) exposures. The number of cells per mL was determined by optical density measurements at 750 nm (OD_750_) using UV-visible spectroscopy. The chlorophyll concentration was determined spectrophotometrically in 95% (v/v) ethanol.

### PEGylation of *Escherichia coli*

A single colony of *E. coli* was extracted from a Lysogeny Broth (LB) plate medium and transferred into LB liquid medium and incubated at 37 °C for 24 h. Five millilitres of the washed bacterial cells (OD_600_ = 1.0) were added to PBS solution (pH = 7.4) and 10 mg/mL mPEG-NHS added at room temperature. After 6 h, the PEGylated bacterial cells were thoroughly washed using DI water and centrifugation. The grafting density (*D*; number of PEG chains per square nanometre of cell surface) was calculated by Eq. (1):

1 $$D = \left( {m_1 - m_2} \right)/\left( {M_{\mathrm{PEG}} \times n_{\mathrm{bac}} \times S_{\mathrm{bac}}} \right),$$

where *m*_*1*_ and *m*_*2*_ were the respective weights of PEG before and after grafting reaction, *M*_PEG_ is the molecular weight of PEG, *n*_bac_ the number of reacted *E. coli* cells *and S*_bac_ was the superficial area of single *E. coli*.

### Cell viability tests

FDA was dissolved in acetone (5 mg/mL) and 5 μL transferred to a centrifuge tube containing 1 mL of native *Chlorella* cells, *Saccharomyces cerevisiae* (*S. cerevisiae*) cells or fabricated cell-based spheroids. After incubated at room temperature in the dark, the cells or spheroids were washed three times using DI water and then imaged using a fluorescence microscope.

### Preparation of denatured BSA particles

A BSA aqueous solution was dialyzed (dialysis tube, 12–14 kDa MWCO) against deionized water for 2 days to eliminate potential impurities. After freeze drying, the purified BSA was stored at −20 °C for subsequent usage. To efficiently stabilize the w/w dextran-in-PEG emulsions, denatured BSA particles were prepared by heating. In brief, BSA was thoroughly dissolved in deionized water (pH = 2, 3 wt%), filtered (mesh size of 0.22 μm) and then tightly sealed in a sample tube and transferred into an oil bath at 90 °C. Typically, the protein solutions were stirred at 400 rpm for 20 h to produce a dispersion of denatured BSA microparticles, which was then stored at 4 °C for further use. The denatured BSA particles were labelled with the hydrophobic dye Nile Red (10–20 μM) by incubation in the dye solution at 60 °C for 30 min.

### Preparation of w/w dextran-in-PEG emulsion droplets

A total of 120 μL of 160 mg/mL dextran aqueous solution was mixed with 120 μL of an aqueous dispersion of denatured BSA particles (3 wt%) and slowly injected into a vial containing 1.2 mL of 160 mg/mL PEG aqueous solution and a well-sized stirrer over a course of 30 s, followed by full homogenization for several minutes. For green fluorescence labelling of the dextran phase, 20 μL of 1 mg/mL FITC-dextran was supplemented into the dextran solution prior to emulsification. To control the size distribution of the dextran-in-PEG emulsion micro-droplets, the spinning rate of the stirrer was adjusted over the range of 100, 200, 400, 600, 800 and 1000 rpm.

### Fabrication of *Chlorella* cell-containing emulsion droplets

A total of *Chlorella* cells were thoroughly washed with NaCl aqueous solution (0.01 M) and DI water by repeated centrifugation (5000 × *g* for 3 min for three times) and the concentration was adjusted to ~3.3 × 10^9^ cells/mL (solution volume, 120 μL). The cell dispersion was added to 240 μL of an aqueous solution of dextran (120 μL, 160 mg/mL) and denatured BSA particles (120 μL, 3 wt%), and the resulting mixture slowly injected into 1.2 mL of 160 mg/mL PEG solution under stirring (100, 200 and 400 rpm) for several minutes to produce dextran-in-PEG w/w emulsion droplets containing high numbers of captured *Chlorella* cells. The statistical average cell number per droplet (*n*) was calculated using Eq. (2):

2 $$n = \frac{{qcV_1}}{{\frac{{V_2}}{{(4/3)\pi r^3}}}},$$

where *q* was the encapsulation efficiency (*ca*. 1.0); *c* and *V*_1_ were the cell density (cells/mL) and volume (mL) of used algal cells, respectively; *V*_2_ was the volume of dextran phase (mL); and *r* the average radius of the *Chlorella*-entrapped droplets (μm). The calculation gave *n* values of *ca*. 8700 and 180 for *Chlorella*-entrapped droplets prepared with cell densities of 3.3 × 10^9^ or 6.9 × 10^7^ cells/mL, respectively.

### Construction of *Chlorella* and *Chlorella*/*E. coli* multicellular spheroids

We prepared w/w dextran-in-PEG emulsion droplets containing algal or algal/PEGylated bacteria cells (OD_600_ (*E. coli*): OD_750_ (*Chlorella*) = 1:80) as described above at 200 rpm. In both cases, the droplets were slowly transferred into a hyperosmotic PEG aqueous solution (50%, w/w, M_W_ 2000 Da) to induce simultaneously shrinking of the droplets and compaction of the entrapped *Chlorella* or *Chlorella*/*E. coli* cells. The vials were left unstirred and the sedimented spheroids collected by carefully removing the supernatant followed by washing several times using DI water.

### Synthesis of aldehyde-functionalized dextran

We dissolved 2 g of Dextran (M_W_ 70 kDa), 0.96 g of 4-formylbenzoic acid and 0.12 g of 4-(dimethylamino) pyridine (DMAP) in 40 mL of dimethyl sulfoxide (DMSO). Then, 1.2 g of N,N-dicyclohexylcarbodiimide (DCC) were added. The mixture solution was kept stirring at room temperature for 18 h. To obtain the product Dex-CHO, the reaction solution was added into the mixture of ethyl acetate and petroleum ether (volume ratio, 1:9). Dex-CHO was then dissolved in the DI water during which impurities were removed by filtration. Finally, Dex-CHO was obtained through freeze-dried treatment.

### Construction of crosslinked robust *Chlorella*/*E. coli* multicellular spheroids

Algal/PEGylated bacterial cell mixtures (OD_600_ (*E. coli*): OD_750_ (*Chlorella*) = 1: 80, 120 μL) were added to 360 μL of an aqueous solution of dextran (120 μL, 160 mg/mL), denatured BSA particles (120 μL, 3 wt%) and Dex-CHO (120 μL, 2.4 wt%). The w/w emulsion droplets were slowly transferred into a hyperosmotic PEG solution dissolved in 10 mM PBS (pH = 7.4, 50% w/w, M_W_ 2000 Da) and left 20 min to simultaneously induce the shrinking of the droplets, compaction of the entrapped *Chlorella* and *E. coli* cells as well as the crosslinking reaction between Dex-CHO and the BSA particles. The vials were left unstirred and the sedimented spheroids were collected by carefully washing several times using DI water.

### Photosynthetic oxygen/hydrogen production

A fixed volume of free algal cells, algal spheroids or algal/bacterial spheroids (*ca*. 35 mg/L chlorophyll) was transferred to a 10 mL serum bottle (8 mL head air space and 2 mL TAP culture medium) and exposed to a light intensity of 100 μE m^−2^ s^−1^. Samples were shaken throughout at a rate of 150 rpm. Time-dependent changes in oxygen and hydrogen concentrations in the samples were monitored using an Oxygen Detector (AP-B-O_2_-F; capacity, 30% vol; resolution ratio, 0.1% vol) and Hydrogen Detector (AP-B-H_2_-F; capacity, 1000 ppm; resolution ratio, 1 ppm). The rates of gas production were calculated according to the total content of chlorophyll associated with the spheroids.

### In vivo hydrogenase activity

To monitor in vivo hydrogenase activity, typically, 500 μL (17.5 μg chlorophyll content) of a suspension of algal or algal/bacterial cell spheroids was transferred after exposure to daylight for specified times to a 5 mL glass vial, and instantly sparged with argon for 15 min to remove all residual oxygen. The vial was tightly sealed and then incubated at 25 °C for 1 h under daylight (light intensity, 100 μE m^−2^ s^−1^). Samples were shaken throughout at a rate of 150 rpm. The in vivo hydrogenase activity was then calculated.

### Measurement of intracellular ATP

Suspensions of native *Chlorella* cells or *Chlorella* cell-based spheroids were diluted with PBS solution (pH = 7.4) and the intracellular ATP extracted by repeated alternate freezing and thawing cycles. The samples were then centrifuged at 2000 × *g* for 20 min and the supernatant were collected. The concentration of ATP was determined spectrophotometrically using an ATP Elisa Kit (Detect Technical Institute, Shanghai, China).

### Determination of the chlorophyll content

Hundred microlitres of a multicellular spheroid suspension was transferred into a centrifuge tube. After centrifugation, the supernatant solution was removed and 100 μL of ethanol added. The mixture was completely homogenized, and the stirred mixture left for at least 12 h at 4 °C. The mixture was then centrifuged and the chlorophyll concentration in the supernatant spectroscopically determined from the absorbance values at 665 nm (A_665_) and 649 nm (A_649_). The total concentration of chlorophyll (C, μg/mL) was calculated using Eq. (3):

3 $${\mathrm{C}} = 6.1 \times {\mathrm{A}}_{665} + 20.4 \times {\mathrm{A}}_{649}$$

### Hydrogen termination by DCMU

The stock solutions of DCMU were prepared by dissolving 0.05 g of DCMU in 20 mL of acetone. For the termination of hydrogen production on the microbial reactors, 40 μL of DCMU solutions were added into the 2 mL of TAP culture medium with a final concentration of 200 μM.

### Ethanol generation from *Saccharomyces cerevisiae* spheroids

A single colony of *S. cerevisiae* was extracted from a yeast extract peptone dextrose (YPD) plate medium and transferred into YPD broth liquid medium and incubated at 30 °C for 16 h. The solution was then centrifuged, and the *S. cerevisiae* cells were washed for three times using DI water. Subsequently, encapsulation of the cells into w/w dextran-in-PEG emulsions as well as the fabrication of the multicellular spheroids was conducted in a way similar to that described above for *Chlorella* cells. *S. cerevisiae* cell-containing droplets or *S. cerevisiae* spheroids were dispersed in 2 mL of DI water and incubated at 30 °C for 12 h. Thereafter, the suspension was centrifuged, and the level of ethanol in the supernatant was determined by FTIR spectroscopy.

### Reporting summary

Further information on research design is available in the Nature Research Reporting Summary linked to this article.

## Supplementary information

Supplementary Information

Peer Review File

Reporting Summary

## Data Availability

Data supporting the findings of this work are available within the paper and its Supplementary Information files. A reporting summary for this Article is available as a Supplementary Information file. The datasets and plant materials generated and analyzed during the current study are available from the corresponding author upon request. Source data are provided with this paper.

## Source data

Source Data
