# Supplementary material for: Photosynthetic hydrogen production by droplet-based microbial micro-reactors under aerobic conditions
Source: Nat Commun. 2020 Nov 25;11:5985. doi: 10.1038/s41467-020-19823-5 (PMC7689460; doi:10.1038/s41467-020-19823-5)
Supplement: Supplementary file 1 — Supplementary Information [file 41467_2020_19823_MOESM1_ESM.pdf]

Photosynthetic hydrogen production by droplet-based microbial micro-reactors under aerobic conditions

Xu *et al.*

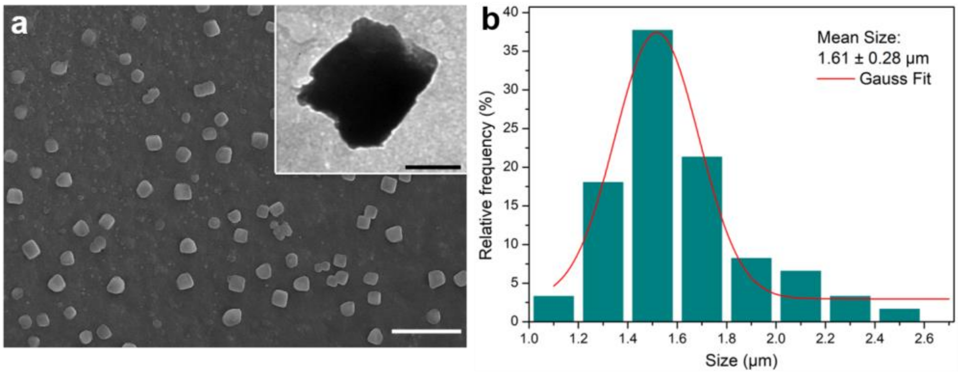

Supplementary Figure 1. Morphology characterization of BSA particles. (a) SEM and TEM (inset) images of BSA microparticles produced after incubating an aqueous BSA solution at pH = 2 and 90 ºC for 20 h; Scale bars, 5 μm and 500 nm (inset), respectively. (b) Corresponding histogram of BSA particle size distribution derived from (a). The single peak fitting was conducted by applying a Gauss curve to the statistical histogram. The mean particle size was 1.6 μm. All relevant experiments were performed independently at least three times with similar results.

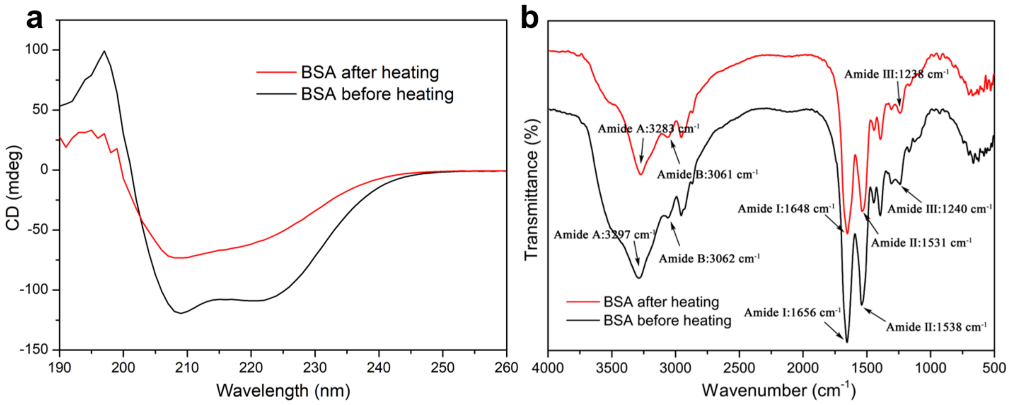

Supplementary Figure 2. **Denaturation of BSA after heating.** CD spectra (a) and FTIR spectra (b) of BSA before and after heating at 90 ºC for 20 h. The CD results indicate that BSA before heating contains 67.7% α-helix, 2.8% β-sheet and 7.0% β-turn, while BSA after heating contains 34.9% α-helix, 20.3% β-sheet and 10.0% β-turn, indicating heat-induced BSA unfolding. This is in agreement with the FTIR spectra of BSA before and after heat treatment, which show Amide A, Amide I and Amide III peaks with different levels of blue shifts. The blue shift of the Amide A band indicates breakage of hydrogen bonds on denaturation. Amide I band features are characteristic of α-helix and the shift to a lower wavenumber indicates a decrease in α-helix content. The Amide III band usually indicates β-sheet and β-turn conformations, and the shift to lower wavenumber demonstrates an increase in the content of β-sheet and β-turn.

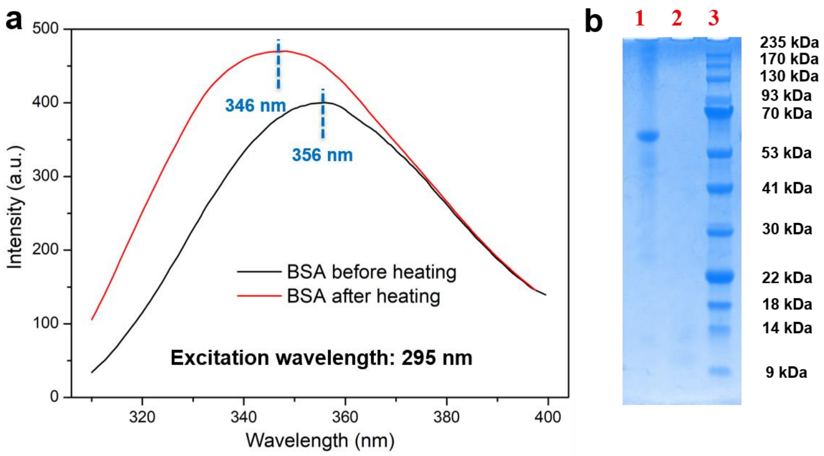

Supplementary Figure 3. **Tryptophan fluorescence spectra (a) and SDS-PAGE profile (b) of BSA before and after heating at 90 °C for 20 h.** Lane 1: native BSA; Lane 2: heated BSA; Lane 3: marker. From (a), the maximum emission wavelength of natural BSA is at 356 nm, while that of BSA heated at 90 °C for 20 h is blue shifted to 346 nm. This result demonstrates that tryptophan residues are blocked in the hydrophobic microenvironment because of denaturation and aggregation of BSA induced by the heat treatment. The aggregation is also confirmed by SDS-PAGE, in which compared to native BSA (Lane 1), heated BSA did not show any characteristic band (Lane 2), indicating the absence of monomeric BSA after heating treatment. All relevant experiments were performed independently at least three times with similar results.

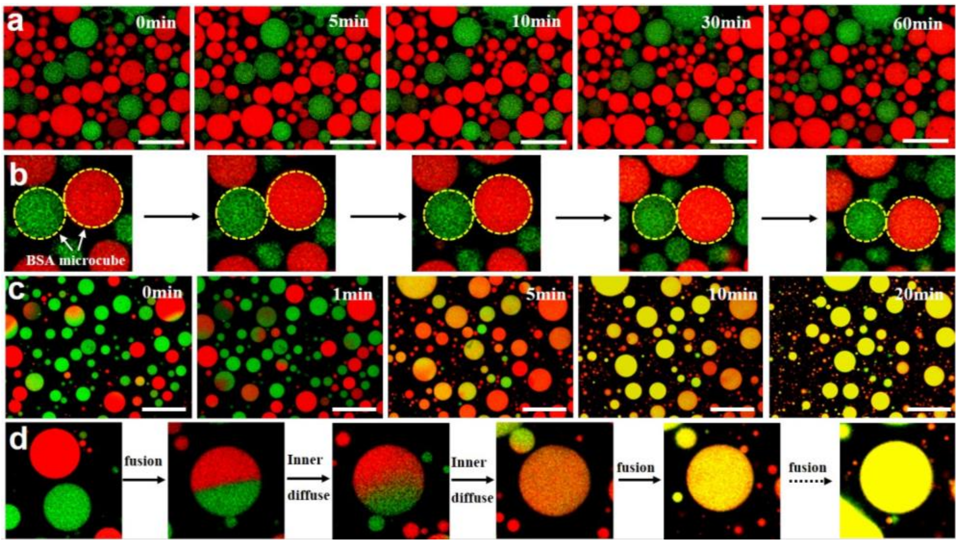

Supplementary Figure 4. **Interfacial stabilization of dextran/PEG w/w emulsions with denatured BSA particles.** Time-sequence of confocal fluorescence microscopy images showing the absence or presence of coalescence for dextran-rich droplets mixed with (a,b) or without (c,d) degraded BSA particles; (b) and (d) show non-fusion or coalescence, respectively, for two typical droplets. All scale bars, 50 μm. Droplets were stained with either RITC-dextran (red fluorescence) and FITC-dextran (green fluorescence). All relevant experiments were performed independently at least three times with similar results.

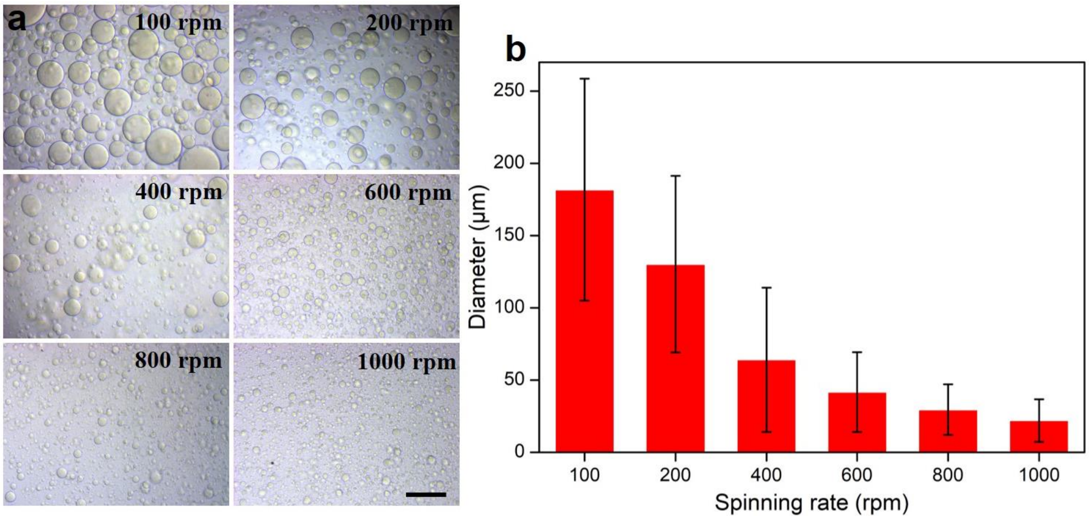

Supplementary Figure 5. Influence of shear force on emulsion droplet size. (a) Representative optical microscopy images of dextran-in-PEG micro-droplets prepared under different shear stresses (from 100, 200, 400, 600, 800 to 1000 rpm). Scale bar, 400 μm. (b) Corresponding diameter distribution histograms of (a). Data are presented as mean values ± SD, error bars indicate standard deviations. All relevant experiments were performed independently at least three times with similar results. Source data underlying Supplementary Figure 5b are provided as a Source Data file.

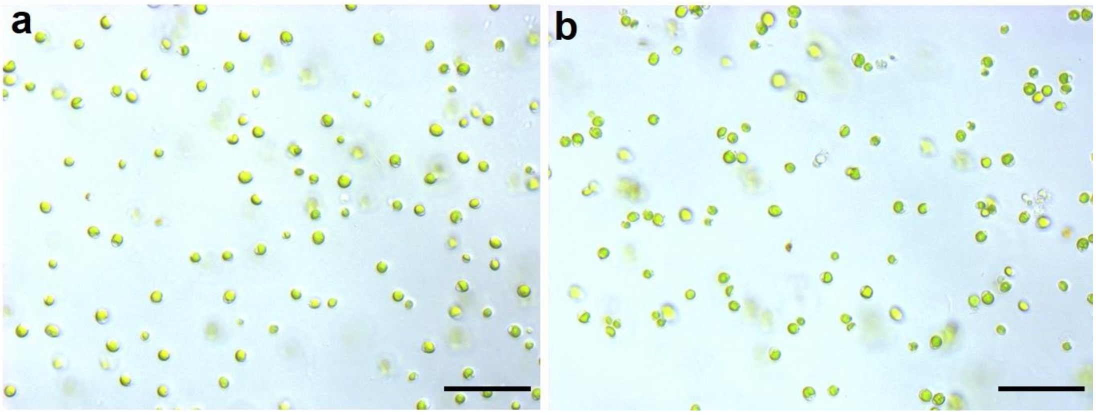

Supplementary Figure 6. **Optical microscopy images of native *Chlorella* cells dispersed in deionized water (a), or when mixed with a suspension of denatured BSA microparticles (3 wt%) (b).** No obvious aggregation is observed in the presence of the emulsion stabilizer. Scale bars, 50 μm. All relevant experiments were performed independently at least three times with similar results.

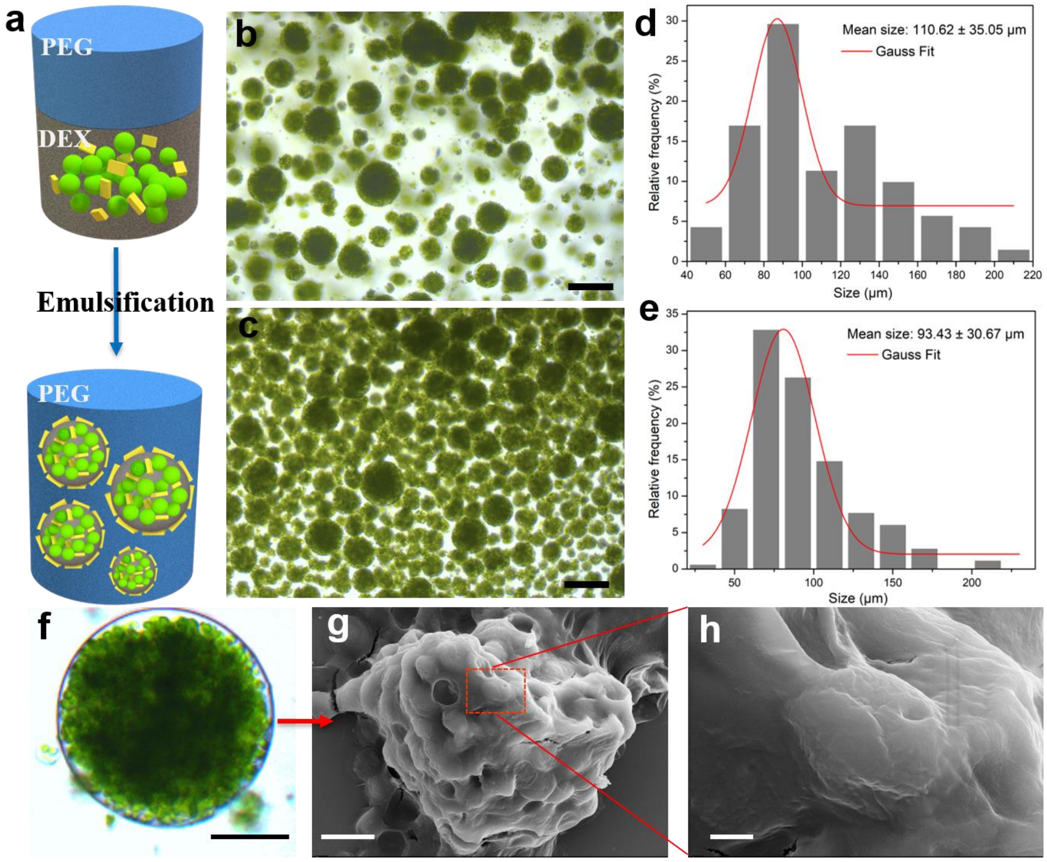

Supplementary Figure 7. Stability of *Chlorella*-loaded w/w emulsions. (a) Schematic illustration showing the process of *Chlorella* cell encapsulation in dextran-in-PEG microdroplets. 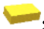, BSA particles; 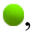, *Chlorella* cell. (b,c) Optical microscopy images of emulsion droplet-entrapped *Chlorella* cells recorded immediately after homogenisation (b), and then after 30 mins (c). Scale bars, 200 μm. (d,e) Corresponding diameter distribution histograms from (b) and (c), respectively. The single peak fitting was conducted by applying a Gauss curve to the statistical histogram. No obvious differences in droplet diameter were observed, indicating that the *Chlorella*-containing micro-droplets were stable. (The slight decrease of the diameter was due to the sediment of smaller droplets onto the bottom of the glass slide). (f) Typical optical microscopy images of *Chlorella* cells entrapped within a single w/w emulsion droplet. Scale bar, 50 μm. (g,h) SEM images of a single *Chlorella*-containing micro-droplet after drying recorded at low (g) and high (h) magnifications; respective scale bars, 10 μm and 1 μm. A continuous outer shell is observed at high magnification. All relevant experiments were performed independently at least three times with similar results.

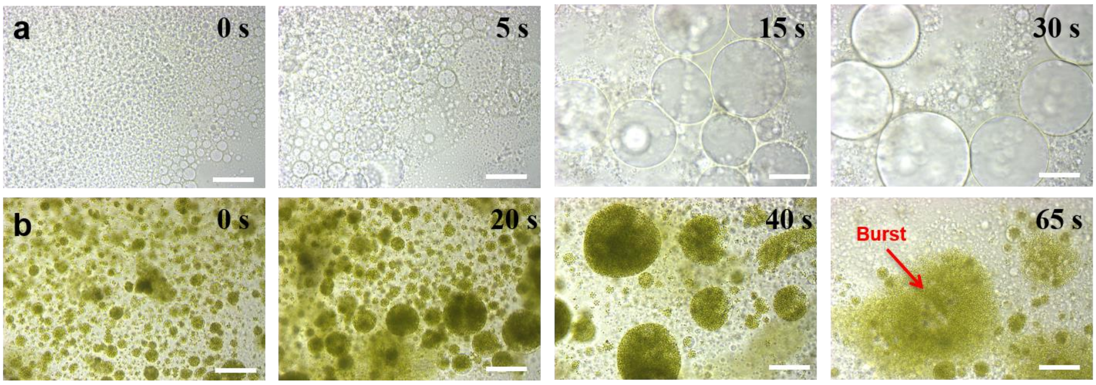

Supplementary Figure 8. Time sequences of optical microscope images of PEG-in-dextran droplets (a) and *Chlorella*-loaded PEG-in-dextran droplets (b) stabilized by BSA particles (3 wt%). Scale bars, 100 μm. The PEG-in-dextran droplets were relatively small and extremely unstable with respect to coalescence. Attempts to encapsulate the *Chlorella* cells within the PEG phase resulted in droplet fusion and release of the algal cells into the continuous dextran phase. All relevant experiments were performed independently at least three times with similar results.

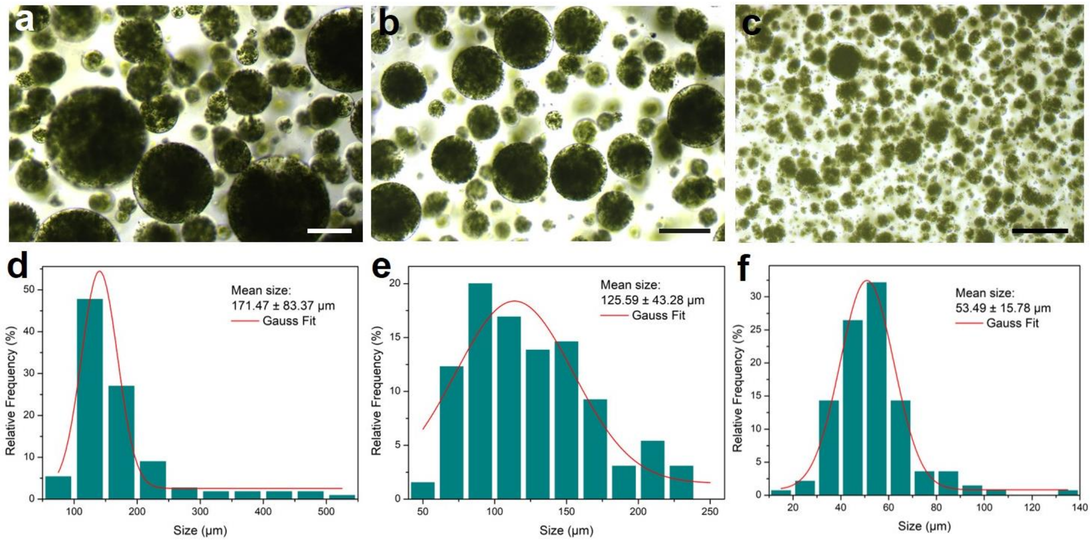

Supplementary Figure 9. **Preparation of *Chlorella* cell-loaded droplets.** (a-c) Representative optical microscopy images showing *Chlorella* cells entrapped within dextran-in-PEG microdroplets formed under spinning rates of 100 rpm (a), 200 rpm (b), 400 rpm (c). (d-f) Corresponding plots of size distribution histograms with Gaussian curve fitting from sufficient statistics; mean sizes, 171, 126 and 53 μm, respectively. All scale bars, 200 μm. All relevant experiments were performed independently at least three times with similar results.

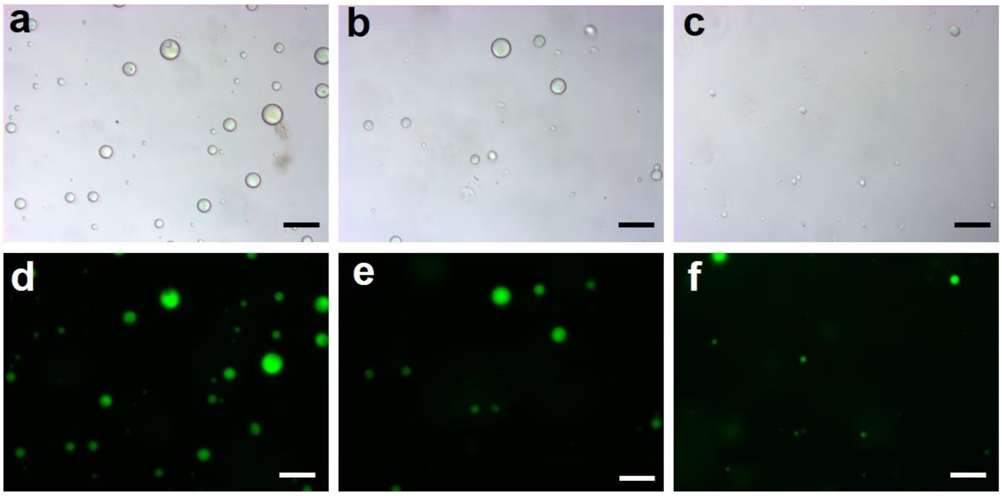

Supplementary Figure 10. **Volume contraction of w/w emulsion droplets after immersion in hyperosmotic PEG solution.** (a-f) Representative optical microscopy images (a-c) and corresponding fluorescence microscopy images (d-f) of dextran-in-PEG micro-droplets after exposure to PEG (50% w/w, MW 2000 Da). Samples were prepared at spinning rates of 100 rpm (a,d), 200 rpm (b,e), 400 rpm (c,f) using FITC-dextran (green fluorescence). All scale bars, 50 µm. All relevant experiments were performed independently at least three times with similar results.

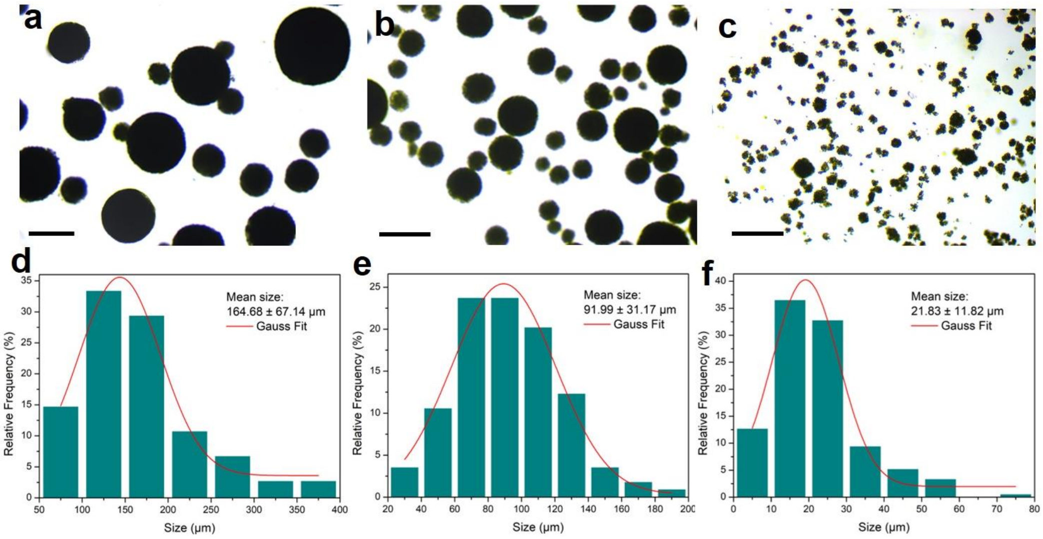

Supplementary Figure 11. **Preparation of *Chlorella* cell-based spheroids.** (a-c) Representative optical microscopy images of spheroids produced after osmotic compression of dextran-in-PEG w/w emulsion micro-droplets prepared at spinning rates of 100 rpm (a), 200 rpm (b) and 400 rpm (c). (d-f) Corresponding plots of size distribution histograms with Gaussian curve fitting from sufficient statistics; mean sizes, 165, 92 and 22 μm, respectively. All scale bars, 200 μm. All relevant experiments were performed independently at least three times with similar results.

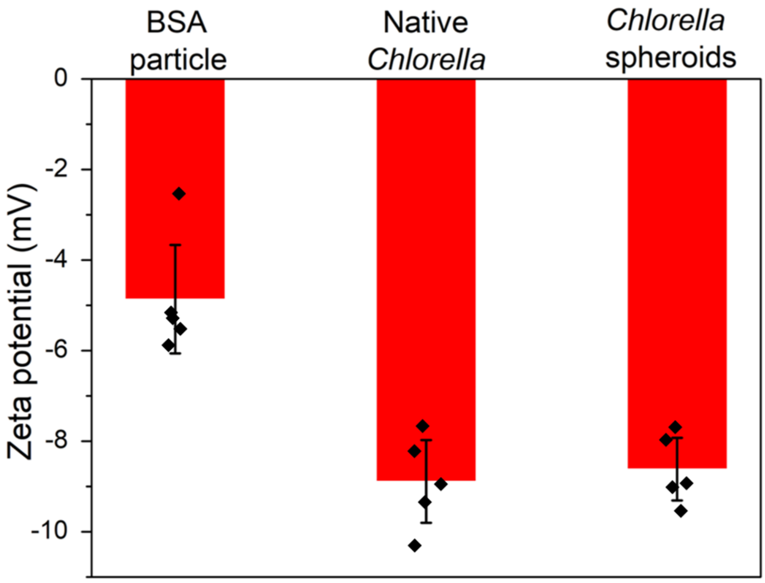

Supplementary Figure 12. **Zeta potential measurements of BSA particles, native *Chlorella* cells and *Chlorella* cell spheroids in PBS buffer (pH = 7.0).** BSA particles: -4.86 mV; native *Chlorella* cells: -8.89 mV; *Chlorella* cell spheroids: -8.62 mV. Compared to the native *Chlorella* cells, a negligible decrease in zeta potential value is observed for the *Chlorella* cell spheroids, indicating that aggregation of the cells during osmotic compression was not dependent on changes in the zeta potential. Data are presented as mean values ± SD, error bars indicate standard deviations (n=5). Source data are provided as a Source Data file.

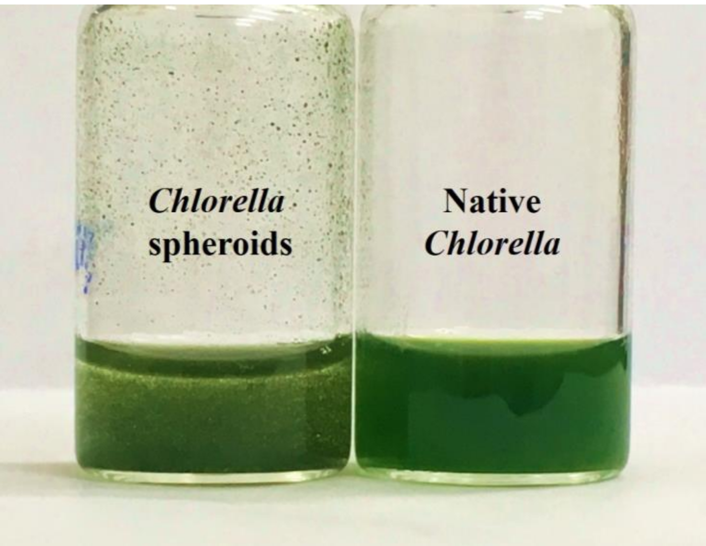

Supplementary Figure 13. **Photographs showing stability of *Chlorella* cell-based spheroids (left) and native *Chlorella* cells (right) dispersed in TAP culture medium.**

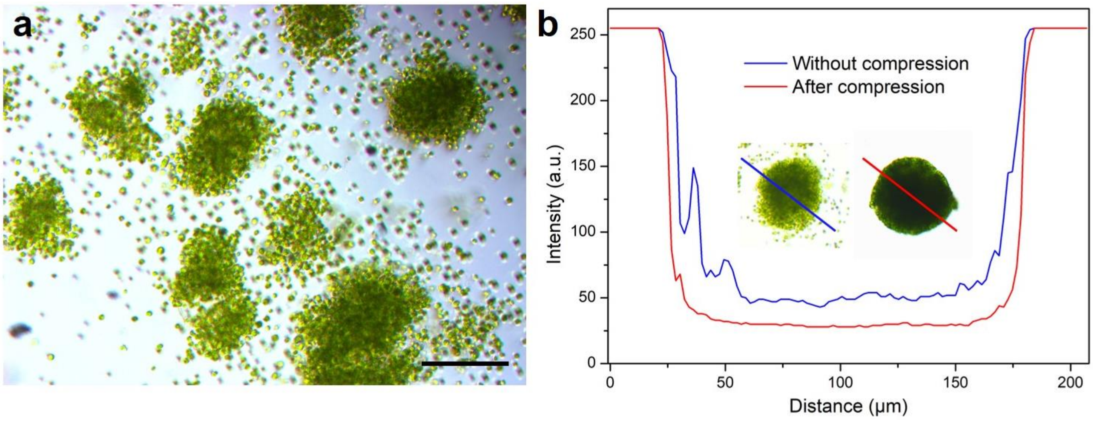

Supplementary Figure 14. ***Chlorella*** **cell clusters prepared without compression.** (a) Optical microscopy image of *Chlorella* cell-loaded dextran-in-PEG emulsion w/w micro-droplets prepared without hyperosmotic compression (spinning rate, 200 rpm) showing disruption of the loosely packed arrangements of algal cells after dilution in water; Scale bar, 100 μm. (b) Brightness line profiles for a single *Chlorella*-containing droplet (no osmotic shrinkage) (blue) and spheroid produce after osmotic compression (red). All relevant experiments were performed independently at least three times with similar results. Source data underlying Supplementary Figure 14b are provided as a Source Data file.

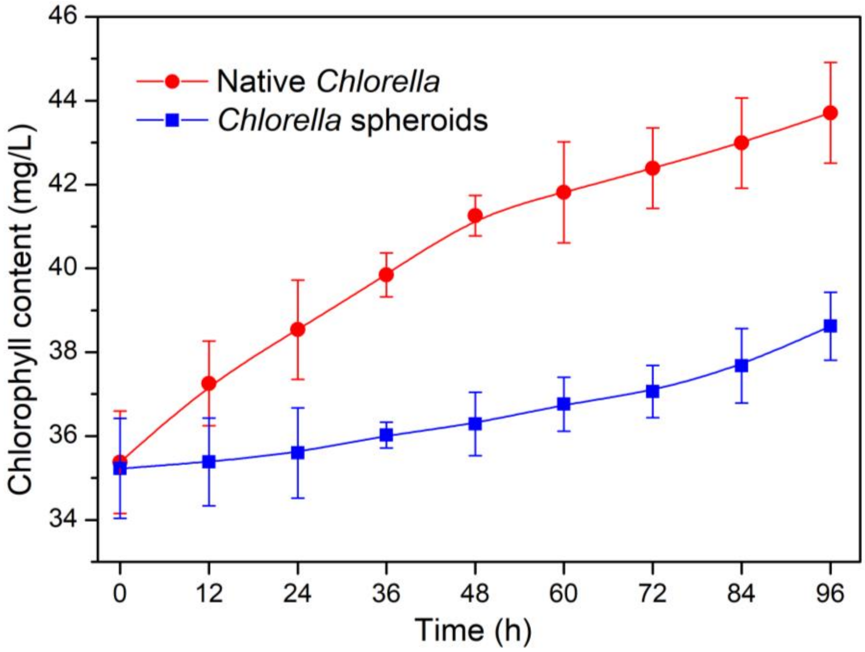

Supplementary Figure 15. **Plot of time-dependent changes in chlorophyll concentration in native *Chlorella* cells (red) and *Chlorella* cell-based spheroids (blue).** Although the growth rate was faster in the native cells, algal cells in the spheroids remained viable and proliferated slowly. Data are presented as mean values ± SD, error bars indicate standard deviations (n=3). Source data are provided as a Source Data file.

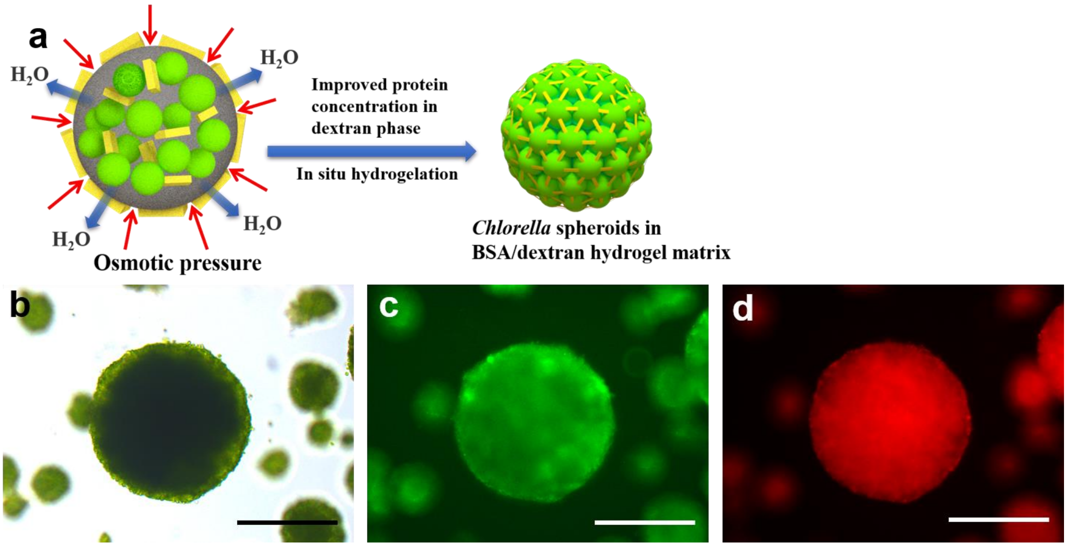

Supplementary Figure 16. Formation of *Chlorella* cell-based spheroids with BSA microparticles in the dextran phase. (a) Schematic diagram showing osmotic compression of the cell-loaded w/w emulsion droplets to produce a closely packed multicellular assembly embedded in a BSA viscous medium (hydrogel). [yellow block symbol], BSA microparticle; [green sphere symbol], *Chlorella* cell. (b) Optical microscopy image of a single *Chlorella* spheroid produced as described in (a) at a spinning rate of 200 rpm. (c,d) Corresponding fluorescence microscopy images of (b) showing BSA particles (green fluorescence) and *Chlorella* cells (chlorophyll, red fluorescence) throughout the spheroids; scale bars, 100 µm. All relevant experiments were performed independently at least three times with similar results.

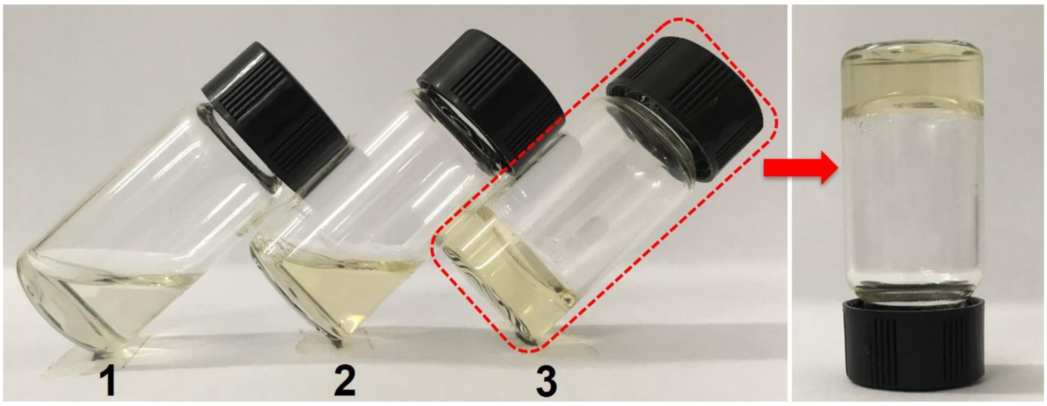

Supplementary Figure 17. **Photographs showing increases in viscosity of BSA-particle solutions prepared at concentrations of 3 wt% (sample 1), 8 wt% (sample 2) and 12 wt% (sample 3) and incubated at 90 °C for 20 h.** A hydrogel is produced at 12 wt%.

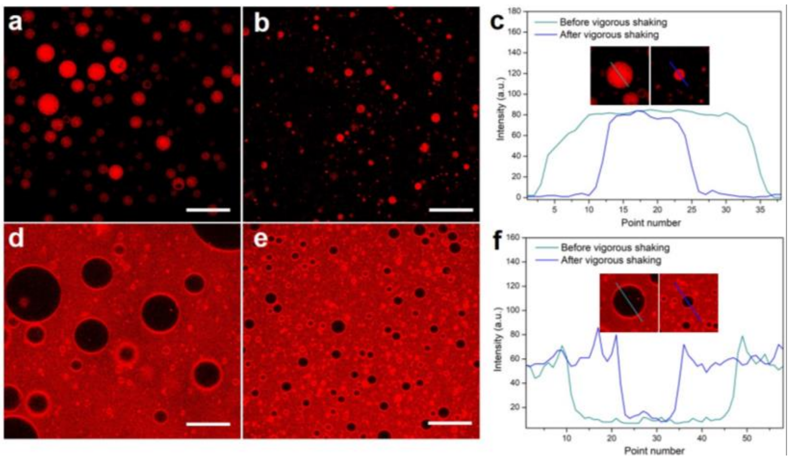

Supplementary Figure 18. **Position of BSA particles in the w/w system.** (a,d) Confocal fluorescence microscopy images showing dextran-in-PEG droplets stabilized by denatured BSA microparticles particles added to the dextran phase (a) or to the PEG phase prior to emulsification (d). (b,e) Corresponding confocal fluorescence microscopy images after vigorous shaking of (a) and (d), respectively. (c,f) Fluorescence intensity line analysis of dextran-in-PEG droplets from (a,b) and (d,e), respectively. The denatured BSA particles were labeled by Nile Red staining (red fluorescence). The denatured BSA particles remained principally within their pre-emulsification phase and did not transfer between the dextran and PEG phases. All scale bars, 50 μm. All relevant experiments were performed independently at least three times with similar results. Source data underlying Supplementary Figure 18c and 18f are provided as a Source Data file.

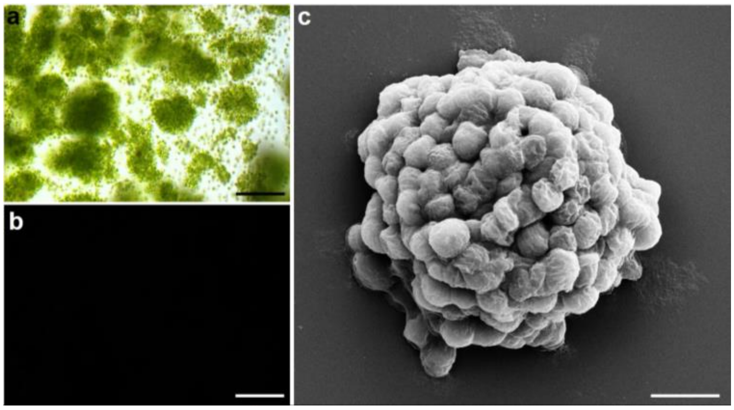

Supplementary Figure 19. **Characterization of *Chlorella* spheroids generated by BSA in PEG phase.** (a) Optical microscopy image of *Chlorella* cell-based spheroids generated from the hyperosmotic compression of dextran-in-PEG droplets stabilized by denatured FITC-labelled BSA particles (green fluorescence) initially added to the PEG phase (spinning rate, 200 rpm). Note the partially disassembly of the algal cell assemblies. scale bar, 100 μm. (b) Corresponding fluorescence microscopy image; the absence of green fluorescence confirmed the absence of the BSA particles within the spheroids; scale bar, 100 μm. (c) SEM image of a single *Chlorella* multicellular spheroid; scale bar, 10 μm. All relevant experiments were performed independently at least three times with similar results.

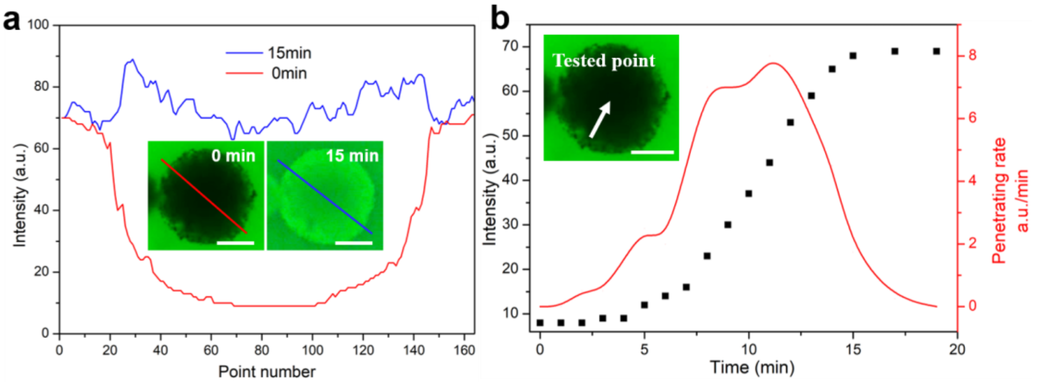

Supplementary Figure 20. Dye diffusion within the *Chlorella* spheroids. (a) Fluorescence intensity line analysis curve across a single spheroid before and 15 min after addition of fluorescein showing slow penetration of the fluorescein molecule into the cluster of closely packed algal cells. Scale bars, 30 µm. (b) Fluorescence penetrating profile at the centre of the tested *Chlorella* spheroid immersed in fluorescein solution. Scale bar, 30 µm. All relevant experiments were performed independently at least three times with similar results. Source data are provided as a Source Data file.

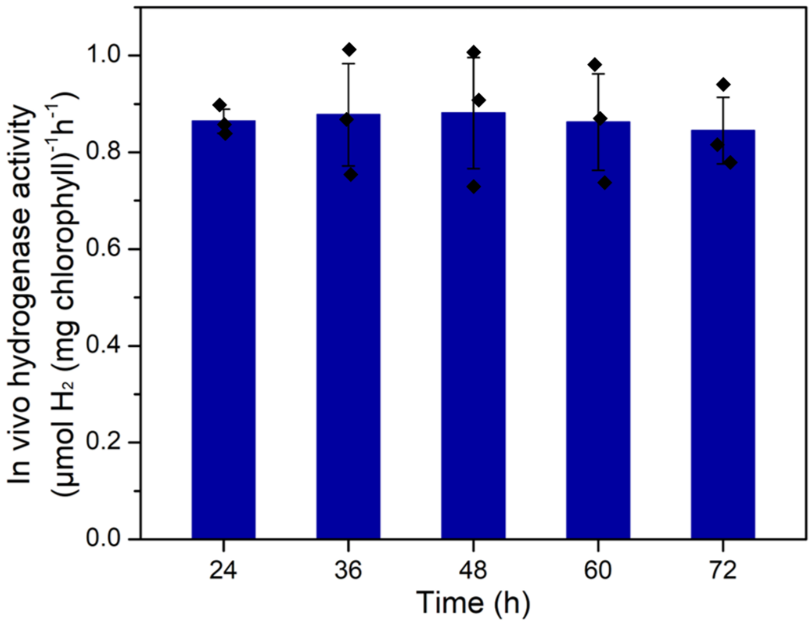

Supplementary Figure 21. ***In vivo* hydrogenase activity measurements of *Chlorella* multicellular spheroids at different time periods under anaerobic conditions (after purging with argon).** A constant high activity of *ca.* 0.86 µmol H_2 (mg chlorophyll)$^{-1}$ h^{-1} was observed over a period of 72 h. Data are presented as mean values ± SD, error bars indicate standard deviations (n=3). Source data are provided as a Source Data file.

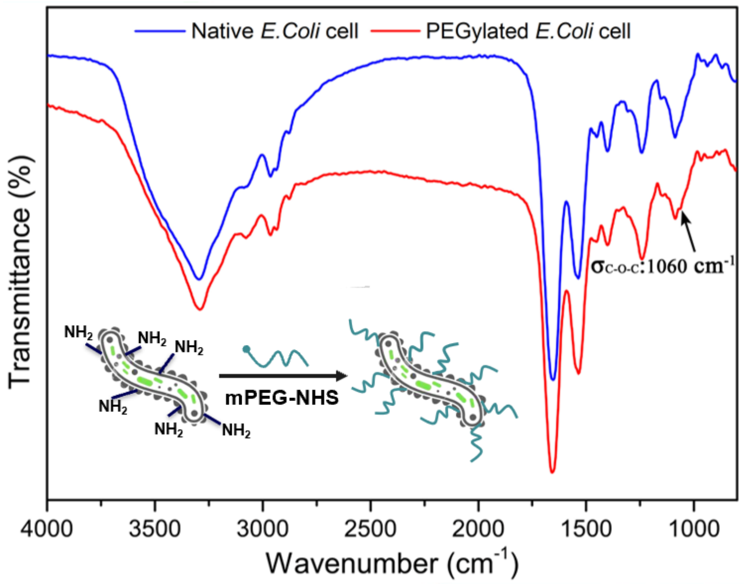

Supplementary Figure 22. **FTIR spectra of dried samples of native (blue curve) and PEGylated (red curve) *E. coli* cells.** Compared to native *E. coli*, the characteristic peak at 1060 cm^{-1} corresponding to the bending C-O-C is sharper in the spectrum of PEGylated *E. coli*, indicating successful conjugation of PEG chains onto the external surface of the bacterial cells. The grafting density was approximately one PEG chain per nm^2 *E. coli* surface.

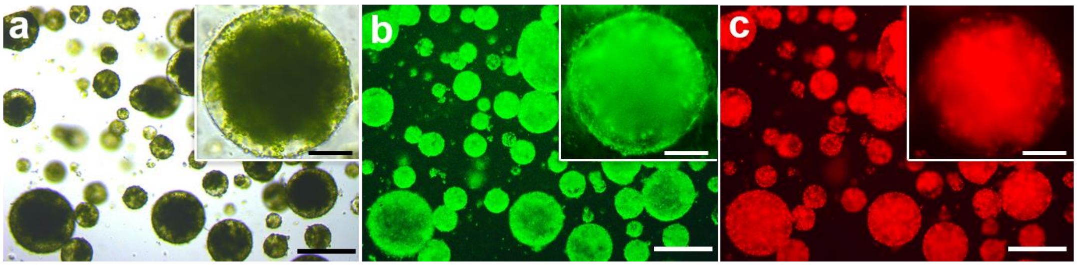

Supplementary Figure 23. Characterization of *Chlorella*/*E. coli* cells-loaded droplet. Optical (a) and fluorescence microscopy images (b,c) of populations of dextran-in-PEG microdroplets entrapped with *Chlorella* cells and PEGylated *E. coli* cells (insets show individual cell-containing droplets). Green fluorescence is from FDA staining of live *Chlorella* cells; Red fluorescence is from chlorophyll; scale bars, 200 μm and 50 μm (insets). All relevant experiments were performed independently at least three times with similar results.

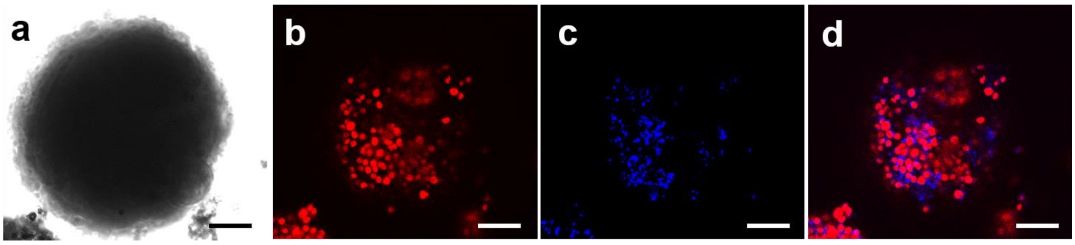

Supplementary Figure 24. Distribution of *Chlorella* and non-PEGylated *E. coli* within the spheroids. (a) Confocal bright field image of a single *Chlorella*/*E. coli* multicellular bacterial spheroid prepared from algal cells and non-PEGylated *E. Coli*. (b-c) Corresponding red (b) and blue (c) confocal fluorescence images. *E. coli* cells are labelled with Atto425 (blue fluorescence) and *Chlorella* cells (red fluorescence, chlorophyll) (d) Overlay image of (b) and (c) showing the homogeneous distribution of *Chlorella* and *E. coli*. Scale bars, 25 μm. All relevant experiments were performed independently at least three times with similar results.

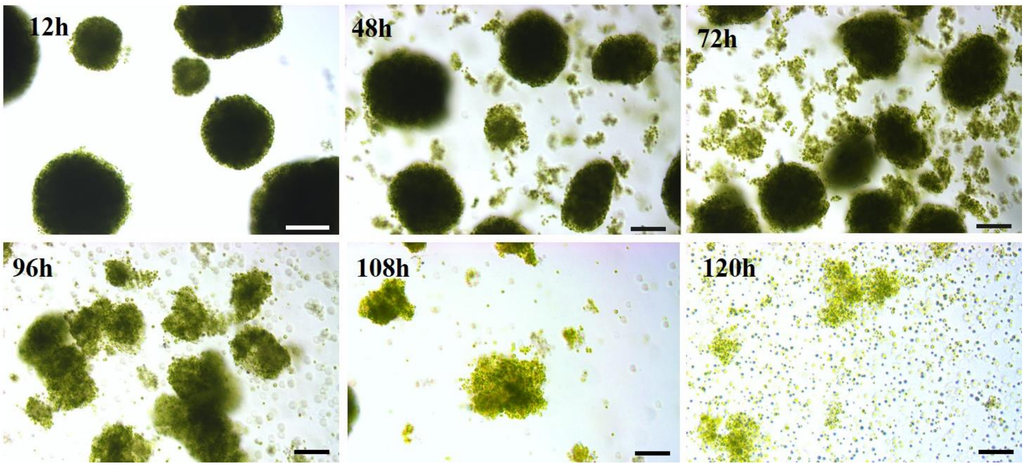

Supplementary Figure 25. **Optical microscopy images showing the gradual time-dependent disintegration of *Chlorella* spheroids undergoing continuous photosynthesis in TAP culture medium**. Scale bars, 50 µm. All relevant experiments were performed independently at least three times with similar results.

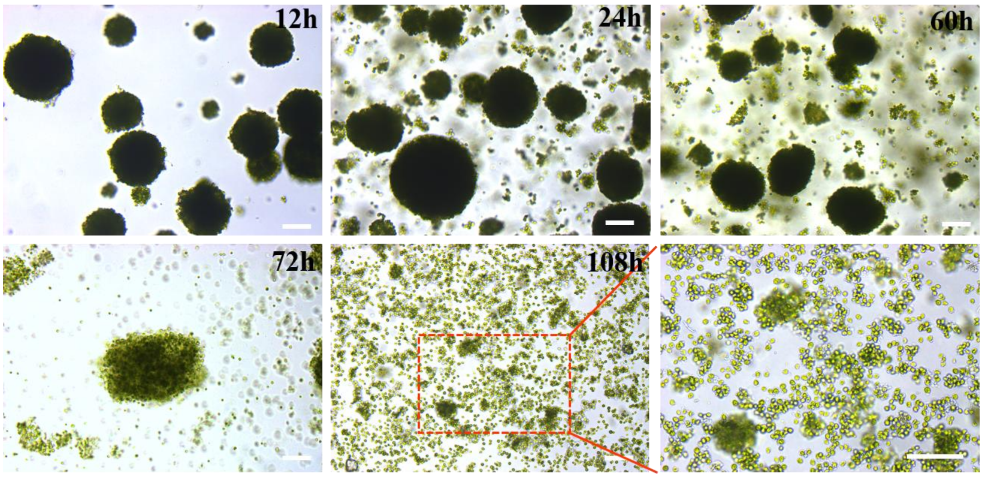

Supplementary Figure 26. **Optical microscopy images showing the gradual time-dependent disintegration of *Chlorella*/*E. coli* hybrid spheroids undergoing continuous photosynthesis in TAP culture medium.** Scale bars, 50 μm. All relevant experiments were performed independently at least three times with similar results.

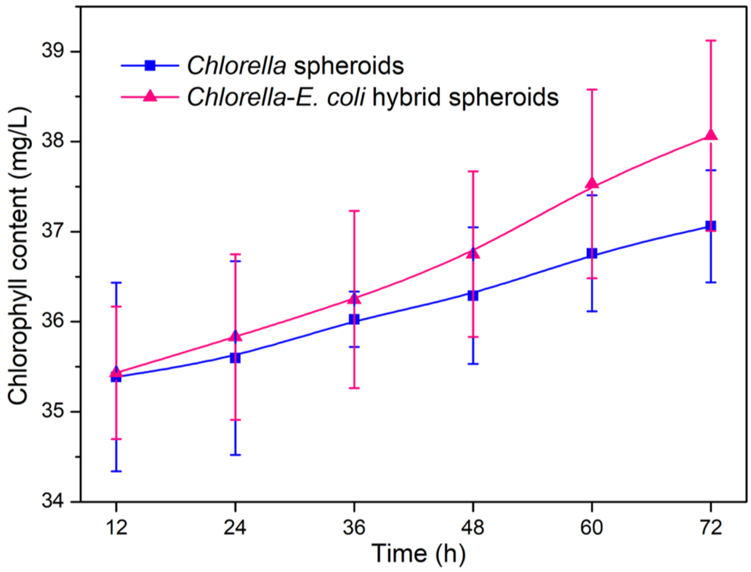

Supplementary Figure 27. Plot of time-dependent changes in chlorophyll concentration in *Chlorella* cell-based spheroids (blue) and *Chlorella*/*E. coli* hybrid spheroids (pink). Data are presented as mean values ± SD, error bars indicate standard deviations (n=3). Source data are provided as a Source Data file.

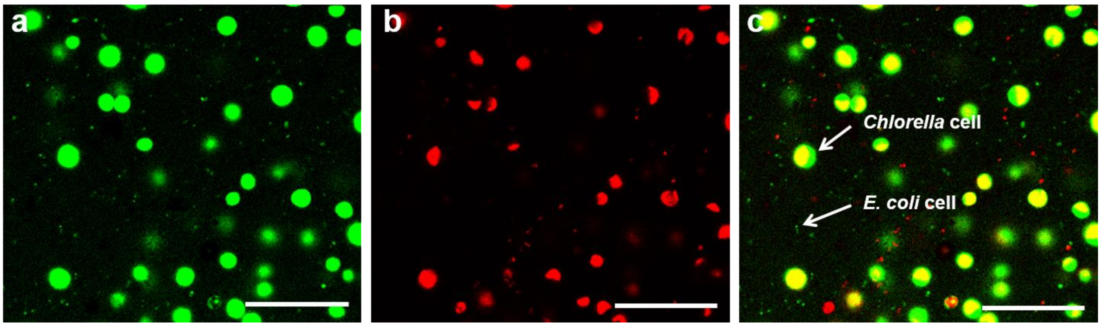

Supplementary Figure 28. **Cell viability after disassembly of *Chlorella*/*E. coli* hybrid spheroids.** Green (a), red (b) and overlay (c) confocal fluorescence images of *Chlorella* and *E. coli* cells after the complete disassembly of *Chlorella*/*E. coli* micro-reactors at 108 h. Green fluorescence is from the hydrolysis of FDA of living cells. Red fluorescence is from intracellular chlorophyll (large objects, *Chlorella* cells) and dead bacterial cells via PI staining (small objects, *E. coli*), respectively. Scale bars, 25 μm. All relevant experiments were performed independently at least three times with similar results.

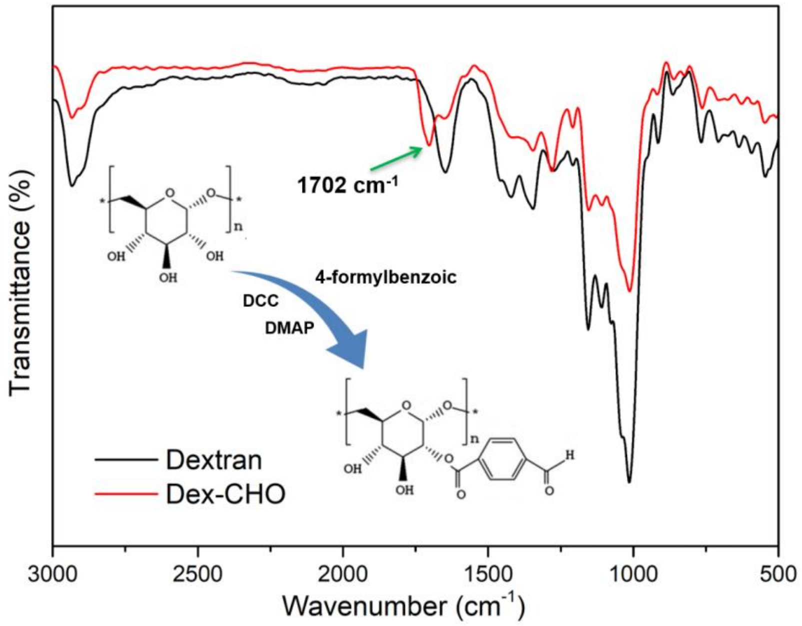

Supplementary Figure 29. FTIR spectra of dextran (black line) and Dex-CHO (red line) confirming successful conjugation between -OH groups in dextran and carboxyl of formyl benzoic acid. The absorption peak is observed at 1702 cm^{-1} (Dex-CHO, formyl benzoic acid, C=O stretch).

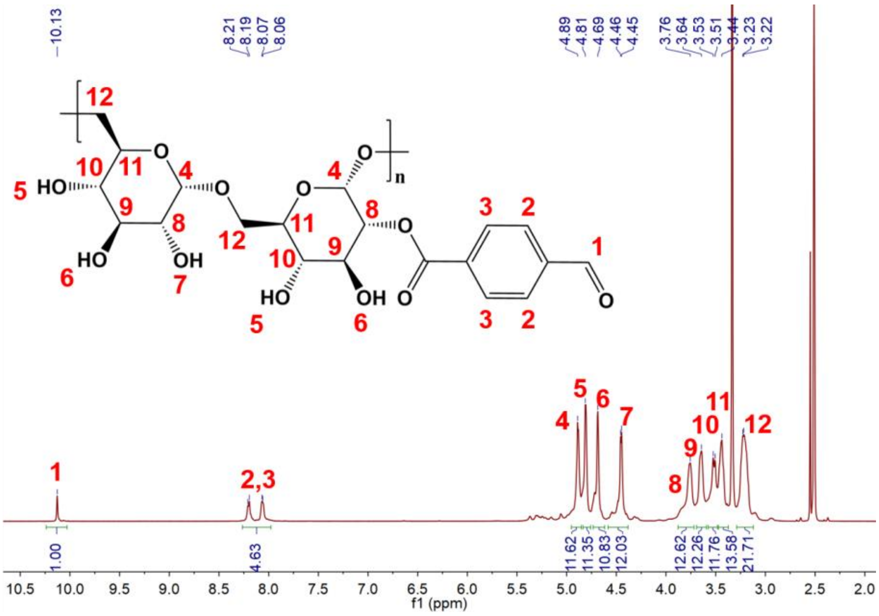

Supplementary Figure 30. ^{1}H-NMR spectrum of Dex-CHO in $(CD_3)_2SO$. Based on the integrated intensity of the aldehyde resonance at 10.13 ppm, every 10 glucose units have one benzyl aldehyde group on average.

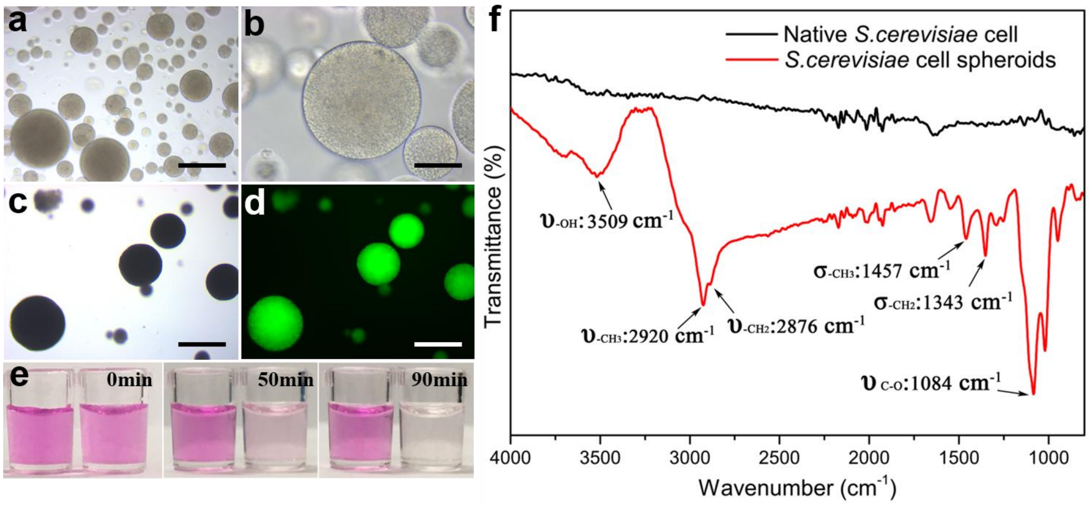

Supplementary Figure 31. **Generation of ethanol from *S. cerevisiae* spheroids.** (a) Optical microscopy images of *S. cerevisiae* cells entrapped within dextran-in-PEG droplets (spinning rate: 200 rpm); scale bar, 200 µm. (b) High magnification image of a single droplet showing presence of large numbers of captured yeast cells; scale bar, 50 µm. (c,d) Optical (c) and fluorescence (d) microscopy images of *S. cerevisiae* spheroids after hyperosmotic compression in PEG solution; scale bars, 200 µm. The *S. cerevisiae*-containing spheroids were stained with FDA. Hydrolysis of FDA by living cells within the spheroids produced high intensity green fluorescence and confirmed the high viability of the yeast cells after encapsulation and hyperosmotic treatment. (e) Time-dependent photographs showing discoloring of an acidic potassium permanganate solution after addition to the supernatant from native *S. cerevisiae* cells (left) or *S. cerevisiae* cell spheroids (right) prepared in culture medium. The faster discoloration observed for the *S. cerevisiae* multicellular spheroids was due to ethanol production associated with anaerobic respiration of the *S. cerevisiae* cells within the oxygen depleted micro-niche produced within the centre of the spheroids. (f) FTIR spectra for the supernatant obtained from a culture medium containing native *S. cerevisiae* cells (black plot) or *S. cerevisiae* multicellular spheroids (red plot). Characteristic peaks at 3509 cm^{-1}, 2920 cm^{-1}, 2876 cm^{-1} and 1084 cm^{-1} corresponding to stretching vibrations of -OH, $-CH_3$, $-CH_2$ and -C-O, respectively, along with bending vibrations of $-CH_3$ and $-CH_2$ at 1457cm^{-1} and 1343cm^{-1}, respectively, confirm the generation of ethanol from the spheroids. In contrast, no absorption peaks for ethanol were detected in the native *S. cerevisiae* cell solution. All relevant experiments were performed independently at least three times with similar results.
