# Supplementary material for: Photosynthetic hydrogen production by droplet-based microbial micro-reactors under aerobic conditions
Source: Nat Commun. 2020 Nov 25;11:5985. doi: 10.1038/s41467-020-19823-5 (PMC7689460; doi:10.1038/s41467-020-19823-5)
Supplement: Supplementary file 2 — Peer Review File [file 41467_2020_19823_MOESM2_ESM.pdf]

Reviewers' comments:
Reviewer #1 (Remarks to the Author):

This work describes the use of an aqueous two phase system-based microdroplets to culture algal cells or algal/bacterial cell communities to enable photosynthesis under daylight. In particular, by co-culturing algal cells and bacterial cells in core-shell geometry, it is shown that hydrogen production can be enhanced. The work demonstrates a clever approach to develop aqueous two-phase system-based microniches with dual functionality to enable photobiological production of hydrogen. Overall, this is a timely work that combines a unique multiphasic system with synergism between two types of biological cells to achieve a potentially useful function of hydrogen production. While the results are quite interesting and novel, there are several questions that must be addressed:
1. What is the efficiency of the system? How does it compared to other synthetic systems?
2. Why do the algal cells segregate to the dextran-rich dispersed phase? Is there specific interactions between dextran and the cell? What happens if PEG-rich phase is the dispersed phase.
3. Could hydrogen production be enhanced by simply adding the bacterial cell to the external PEG-rich phase? Such an experiment must be run as a control to demonstrate that localization of bacteria at the water/water interface.
4. Does the PEGylated bacteria adsorb to the interface in the absence of algal cells in the core of the disperse phase?
5. One critical shortcoming of this approach is that the microbial bioreactor becomes ineffective after 5 days. What are the potential strategies to make hydrogen production a continuous process? I think this is an extremely important point since a lot of the synthetic systems can operate continuously.

Reviewer #2 (Remarks to the Author):

The study exploited utilization of water-in-water dextran-in-PEG emulsion micro-droplets for encapsulating microalga Chlorella pyrenoidosa or assembling Chlorella/E.coli for the photosynthetic production of hydrogen under air. I am not convinced that the study shows enough novelty for publication in Nature Communications. I have the following major concerns, and I recommend rejection of the manuscript in its current form.
1. Developing stable water-in-water dextran-in-PEG emulsion micro-droplets is not anything new. As early as in 2016, Song et al. (Nature Communications, 7:12934, DOI: 10.1038/ncomms12934) developed similar technology, i.e., stable water-in-water dextran-in-PEG emulsion system, but this paper was not cited in the current manuscript.
2. The authors claim that a major contribution of the current study is the spatial organization and immobilization of algal or algal/bacterial cells which creates anaerobic niche in the core of the micro-droplets and induces production of hydrogen. While the basic concept has been demonstrated in a previous study (Ref 24), the current study does not show any groundbreaking progress in innovation. Utilizing w/w immobilization technique improves the

stability of the assembly of cells but disassembly occurs over time and completely disassembled after 5 days (Figure S23). Additionally, the hydrogen production stopped after 72 h (Figure 5h), and the authors did not have appropriate discussion of possible reasons.
3. Page 11, first paragraph. It says the cells at the core were probably shield from the light source. However, hydrogen production consumes energy and there is no mentioning of where this energy comes from if without light/photosynthesis. In Figure 5j, addition of DCMU inhibits PSII and also the hydrogen evolution, but the authors did not explain why. How much DCMU was added into the system? In the section of “Synergistic hydrogen production in Chlorella/E.coli hybrid micro-reactors”, the authors did not explain the E. coli cells respired on what substrate (bottom of page 14). Acetate in the TAP medium? Or excreted organic matters from the Chlorella cells? Did E. coli cells grow and divide at all? In other words, the authors need to clarify what the energy flow is, what the shell cells do and what the cells at the core do and how they are connected. I feel a lot of detailed experiment-based analysis need to be done to answer these questions.

Reviewer #3 (Remarks to the Author):

Overall this is a detailed and interesting study on a novel fabrication method using two-phase aqueous aqueous polymers to entrap algal cells (and other non-photosynthetic cells) into a relatively uniform (20-160 um) particles. Much of the paper especially the supplemental material addresses the methodological approaches such as shearing rpms, shrinking time, etc., Although these experiments do indicate the method is relatively well-controlled and although this is important, it seems this could have been published separately in a more methodological journal. In fact, I also think the paper would be complete and significant if it ended at Figure 4 and S19, thus omitting the E. coli cell inclusion work. Although quite well written and complete, there were also details that seem critical yet were not shown. For example, how denatured was the BSA from the heat treatment this could have been explored using CD, BN-PAGE, even tryptophan fluorescence. It was not even clear that the BSA
needed to be denatured?

It was a bit disappointing to see that even after optimization, that hydrogen production stopped after ~72 hours and then the particles “dissolved” after five days. One would hope the “living” nature of the Chlorella cells would extend this activity longer than the in vitro PSI hydrogen evolution yields shown by others such as the Golbeck lab and in reference 26. These researchers have demonstrated higher yield 5.5 mmol H2/h/mg chlorophyll and a sustained activity for over 85 days? It would be very interesting to see the novel fabrication methods demonstrated here could be applied to include the incorporation of isolated PSI and a catalysis such as the platinum nanospheres or even a Ni/Fe hydrogenase. The integration of enzymatic oxygen scavenging methods (glucose oxidase and catalase) could also make this microsphere particle method compatible with more open environmental applications where anaerobiosis might not be feasible. Overall, it is an interesting and well

done study, that may inspire other similar approaches in the field.

Minor points/suggestions:

- On Figure 2- it was unclear if the Red fluorescence on Panel 2K was the Chl autofluorescence or the fluorescence from the Nile Red from the denatured BSA?

- It is also surprising that the algal cells seem somewhat clumped together and only on the interior? Is this representative or simply one observations. More images would be preferred or possibly the use of flow cytometry to measure the number and distribution using the Slit-scan feature

- With the low cell density (6.9 x 107) there are only a few cells per droplet (I would guess ~20, yet this should be known with statistics using either LSCM or FACS) . However, when you make the droplets using high cell density (3.3 x109) the number of cells goes up to 9000. This is a very large increase 450 times more yet only a 50-fold increase in cell density? This suggests some type of aggregation or algal clustering? Although this has been somewhat systematically studied in the Fig. S3-S9, there are still somewhat inconsistent results. For example, Fig. 3 with Fig. S7. The hyperosmotic shrinking seems to be very different This would be interesting to explore more systematically.

- Assuming diameter of 171 um before hyperosmotic shrinking (~1,964,000 um3) but after the diameter has only reduced to 162 with a volume of 1,764,000 um3. This is only a 11% reduction not the 50% shown in Fig. 3b.

- Figures 3E and F seem redundant to those images shown in Figure 2 K and I.

- -Explanation of large variation of size of their droplets? Is there an issue with reproducibility/controllability of the fabrication?

- -With the photosynthetic Chlorella cells in the interior of these micro-reactors, are there any shading effects that are noted, or heterogeneity in productivity going deeper into these droplets?

- -I was hoping to get more of a broad context in the conclusion to get an idea of where to place this study in comparison to previous work on biomass production by whole cells.

- In trying to explain the lack of hydrogen production in the 22 um sized spheroids as compared to the 92 um, the authors claim that this is due to " to the increased number of algal cells in the aerobic surface regions". Since the particle size is smaller and the surface area much lower, I think the authors actually mean an increased percentage of Algal cells are in an aerobic environment and/or there is a drastic loss of cells in the anaerobic environment. The number of cells is certainly overall less (not more as stated) in the 22 um vs the 92 um.

- On page 12, It is also interesting that the hydrogen yield decreased when they go from 92-165 um, this may be due to the shading as suggested yet this might be a key area for further optimization. It might also be interesting to incorporate antennae reduced mutants to see if increasing the anaerobic environment while also increase light penetrance could further boost the hydrogen yield.

Responses to Reviewer #1:

This work describes the use of an aqueous two phases system-based microdroplets to culture algal cells or algal/bacterial cell communities to enable photosynthesis under daylight. In particular, by co-culturing algal cells and bacterial cells in core-shell geometry, it is shown that hydrogen production can be enhanced. The work demonstrates a clever approach to develop aqueous two-phase system-based microniches with dual functionality to enable photobiological production of hydrogen. Overall, this is a timely work that combines a unique multiphasic system with synergism between two types of biological cells to achieve a potentially useful function of hydrogen production.

Response: We appreciate the reviewer's positive and valuable comments on the scientific merit of our work.

Specific questions:

1. What is the efficiency of the system? How does it compared to other synthetic systems?

Response: The efficiency of the system is shown in two aspects: the preparation process of the microbial micro-reactors and hydrogen production rate of the system. For the former, during the preparation of the microbial spheroids, almost all the *Chlorella* or *E. coli* cells could be encapsulated into the droplets, and the preparation efficiency of our droplet-based microbial micro-reactors is therefore very high. Thus, this is a high throughput method and is suitable for preparing large quantities of microbial spheroids. As for the latter aspect, the hydrogen production rate of the microbial reactor system (*Chlorella*/*E. coli* hybrid spheroids) is 0.45 μmol H_2 (mg chlorophyll)$^{-1}$ h^{-1}. In comparison with other biology-engineered algal cell-based hydrogen production systems, our micro-reactor system exhibited nearly 1.7-fold enhancement compared to that when *Chlamydomonas reinhardtii* Cr849 algal cells and *Pseudomonas* bacteria were co-cultured in sulfur-deprived TAP medium (TAP-S) (*Int. J. Hydrogen Energy*., **2013,** 38, 10779-10787), and at least 1.3-fold enhancement compared to that when pure *Chlamydomonas reinhardtii* cc124 was cultured in TAP-S medium (*Green Chem.*, **2014,** 16, 4716-4727). In addition, our micro-reactor system showed 1.3 and 1.4-fold rate enhancements, respectively, when compared to systems based on silicification-induced algal cell aggregation (0.35 μmol H_2 (mg chlorophyll)$^{-1}$ h^{-1}, *Angew. Chem. Int. Ed.*, **2015,** 54,

11961-11965), and enzyme-mediated anaerobic encapsulation (0.32 μmol H_2 (mg chlorophyll)$^{-1}$ h^{-1}, *Angew. Chem. Int. Ed.*, **2019,** 58, 3992-3995).

2. Why do the algal cells segregate to the dextran-rich dispersed phase? Is there specific interactions between dextran and the cell? What happens if PEG-rich phase is the dispersed phase.

Response: In agreement with our work, previous studies have shown that biomacromolecules and colloidal objects such as yeast cells and human/animal cells are strongly inclined to disperse in the dextran-enriched phase of demixed aqueous two-phase systems (*ACS Macro Lett*., **2017,** 6, 679-683; *Mater. Horiz*., **2017,** 4, 1196-1200). The phase selectivity has been generally attributed to the increased polarity of dextran compared with PEG as well as the branched vs linear/flexible molecular confirmations (*Front. Chem.*, **2019,** 7:44).

In response to the Reviewer's question, we undertook a new control experiment in which a dispersed PEG phase was used for the encapsulation of *Chlorella* cells, by interchanging the composition concentrations of dextran and PEG. In detail, the *Chlorella* cell dispersion (1.2×10^8 cells/mL, 120 μL) was added to 240 μL of the aqueous solution of PEG (120 μL, 160 mg/mL) and denatured BSA particles (120 μL, 3 wt%), and the resulting mixture slowly injected into 1.2 mL of 160 mg/mL dextran solution under stirring (100 rpm) for several minutes. Although PEG-in-dextran droplets could be prepared with BSA particles, the droplets were relatively small and extremely unstable such that they rapidly coalesced (**new Figure S8**). When *Chlorella* cells were encapsulated into the PEG phase, the droplets fused with each other immediately and released the *Chlorella* cells into the dextran phase; in contrast, both the empty and cell-loaded dextran-in-PEG droplets were much more stable (**Figure S4** and **Figure S7)**.

The following text has been added on page 6 of the revised manuscript:

"In contrast, no stable cell-loaded emulsion droplets were produced when PEG was used as the loading phase (**Figure S8**)."

The following new Figure has been added as Figure S8 in the SI.

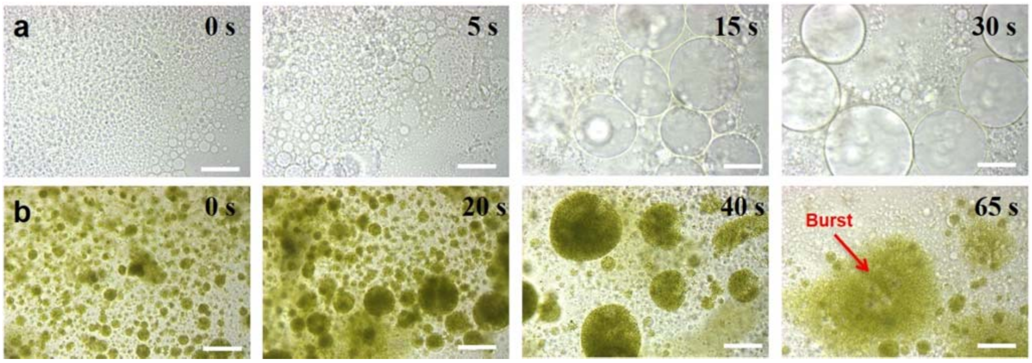

New Figure S8. Time sequences of optical microscope images of PEG-in-dextran droplets (a) and *Chlorella*-loaded PEG-in-dextran droplets (b) stabilized by BSA particles (3 wt%). Scale bars, 100 µm. The PEG-in-dextran droplets were relatively small and extremely unstable with respect to coalescence. Attempts to encapsulate the *Chlorella* cells within the PEG phase resulted in droplet fusion and release of the algal cells into the continuous dextran phase.

3. Could hydrogen production be enhanced by simply adding the bacterial cell to the external PEG-rich phase? Such an experiment must be run as a control to demonstrate that localization of bacteria at the water/water interface.

Response: Many thanks for the valuable suggestion. We performed control experiments in which the bacterial cells were added into the external PEG-rich phase. In the absence of the *Chlorella* spheroids, no hydrogen production was observed. On addition of the free bacterial cells into the external PEG-rich phase, hydrogen production was observed in the presence of *Chlorella* spheroids in the preformed dextran-enriched droplets. However, no enhanced hydrogen production was observed confirming that localization of the bacterial cells at the droplet interface by hyperosmotic shrinkage of assembled multicellular hybrid droplets played a key role in the enhancement of hydrogen generation. All the experiments were performed under the same conditions using the same initial amount of free *E. coli* cells (OD_{600} (*E. Coli*) : OD_{750} (*Chlorella*) = 1 : 80) in the system. Hydrogen yields after 96 h were 1.52 and 1.53 µmol for *Chlorella* spheroids with added *E. coli* cells, and *Chlorella* spheroids alone, respectively.

New control experiments have now been included in Figure 5h, and new text on pages 16 added as follows*:* "In contrast, mixtures of the *Chlorella* spheroids along with free *E. coli* cells showed no enhanced photosynthetic hydrogen production when compared with yields observed for aqueous suspensions of *Chlorella* spheroids alone (**Figure 5h** and **Figure 4c**)."

The following revised Figure has been included as Figure 5h:

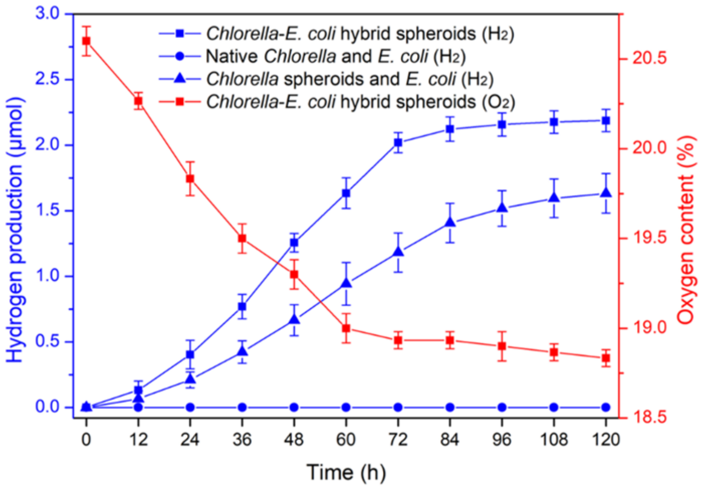

New Figure 5h. Time-dependent measurements of hydrogen concentration in mixed suspensions of native *Chlorella* and *E. coli* cells (blue circles), *Chlorella* spheroids and free *E. coli* cells (blue triangles), and *Chlorella*/*E. coli* multicellular spheroids (blue squares); corresponding decrease in oxygen content for the algal/bacterial cell spheroids is also shown (red squares). All samples were in sealed vials and exposed to daylight at an intensity of 100 μE m^{-2} s^{-1}. Error bars indicate standard deviations (n=3).

4. Does the PEGylated bacteria adsorb to the interface in the absence of algal cells in the core of the disperse phase?

Response: In the absence of algal cells, PEGylated *E. coli* did not strongly absorb at the interface of the two aqueous phases. A spatial distribution of *Chlorella* cells predominantly within the core domain and bacterial cells in the peripheral layer was only obtained when both cell types were mixed in the dextran. As the native *Chlorella* cells were preferentially concentrated in the dextran phase, crowding effects also facilitated the specific location of the PEG-modified *E. coli* at the w/w interface.

5. One critical shortcoming of this approach is that the microbial bioreactor becomes ineffective after 5 days. What are the potential strategies to make hydrogen production a continuous process? I think this is an extremely important point since a lot of the synthetic systems can operate continuously.

Response: Many thanks for the comments. In this study, because of the inherent proliferation of *Chlorella* cells which disrupt the anaerobic microenvironment in the spheroids, the synthesized microbial micro-reactors disassemble and stop hydrogen production after 3 days. In comparison with many reported synthetic or biology-engineered microorganism systems, our studies show that by spatially organizing the living cells we could switch from normal photosynthetic oxygen production to hydrogen production. Therefore, in terms of orchestrating the assembly of different living cells into multicellular ensembles, our approach displays excellent biocompatibility without impairing the viability of the assembled cells; we consider this an important breakthrough.

With regard to the reviewer's suggestion, we undertook further experiments to attempt to prolong hydrogen production by covalently fixing the formed multicellular ensembles to minimize cell proliferation and spheroid disassembly. To do so, aldehyde-functionalized dextran was specially synthesized and used as a cross-linker to further fix the formed multicellular spheroids in the dextran phase. As a consequence, hydrogen production in the optimal bioreactor system (*Chlorella*/*E. coli* hybrid spheroids) was successfully prolonged from 72 to 168 h.

Details of the new experiments have been now incorporated into the "Materials and Methods" section in the SI labelled. The following text has been added on page 2 and page 4/5 of the SI:

Page 2, SI: "… dextran (MW 70 kDa, Pharmacia), 4-formylbenzoic acid (Energy Chemical, 98%), N,N-dicyclohexylcarbodiimide (DCC, Energy Chemical), 4-(dimethylamino) pyridine (DMAP, Sigma)"

Page 4/5, SI: "**Synthesis of aldehyde-functionalized dextran (Dex-CHO).** The functionalized dextran was prepared as follows. Mixtures of dextran (2 g, 0.0285 mmol, MW 70 kDa), 4-formylbenzoic acid (0.96 g, 6.4 mmol), and 4-(dimethylamino) pyridine (DMAP; 0.12 g, 0.984 mmol) were dissolved in 40 mL of dimethyl sulfoxide (DMSO), followed by the addition of N,N-dicyclohexylcarbodiimide (DCC; 1.2 g, 5.83 mmol). The system was stirred at room temperature for 18 h and then the impurity removed by filtration. The Dex-CHO product was obtained as a white solid after precipitation in a mixture of ethyl acetate and petroleum ether with a volume ratio of 1 : 9. The white solid was dissolved in the deionized water, and any insoluble impurities was removed by filtration, and then freeze-dried.

Construction of crosslinked robust *Chlorella*/*E. coli* multicellular spheroids. Algal/PEGylated bacteria cell mixtures (OD_{600} (*E. coli*) : OD_{750} (*Chlorella*) = 1 : 80, 120 µL) were added to 360 µL of an aqueous solution of dextran (120 µL, 160 mg/mL), denatured BSA particles (120 µL, 3 wt%) and Dex-CHO (120 µL, 2.4 wt%). The w/w emulsion droplets were slowly transferred into a hyperosmotic PEG solution dissolved in 10 mM PBS (pH = 7.4, 50 % w/w, MW 2000 Da) and left for 20 min to induce simultaneously shrinking of the droplets, compaction of the entrapped *Chlorella* and *E. coli* cells as well as the crosslinking reaction between Dex-CHO and the BSA particles. The vials were left unstirred and the sedimented spheroids collected by carefully removing the supernatant followed by washing several times using DI water."

The following revised texts have been added to the Abstract and on page 16 of the main manuscript:

Abstract: "…Moreover, the duration of hydrogen production is further prolonged by covalent crosslinking of the droplet microenvironment."

Page 16: "As the *Chlorella* and *Chlorella*/*E. coli* micro-reactors were stabilized only by non-covalent interactions along with *in situ* compaction and hydrogelation of the denatured BSA microparticles during hyperosmotic compression, the spheroids disassembled over time as the cell colonies proliferated (**Figure S25-S27**). As a consequence, hydrogen production

was terminated typically within 72 h even though levels of cell viability after disassembly at 108 h were 68 and 96% for *E. coli* and *Chlorella* cells, respectively (**Figure S28**). To prolong the lifetime of the hybrid bioreactor, we included an aldehyde-functionalized dextran (Dex-CHO) into the w/w dextran-rich emulsion droplets to chemically crosslink the BSA protein hydrogel *in situ*. The resulting spheroids were physically more robust and displayed extended periods of hydrogen production over 168 h (**Figure S29-S31**)."

New data has been added; Figs S26, S27, S28, S29, S30, S31

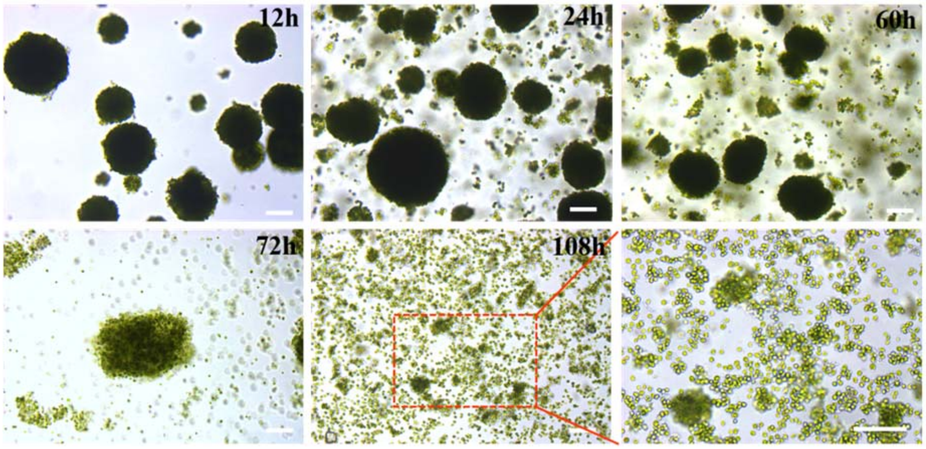

Figure S26. Optical microscopy images showing the gradual time-dependent disintegration of *Chlorella*/*E. coli* hybrid spheroids undergoing continuous photosynthesis in TAP culture medium. Scale bars, 50 µm.

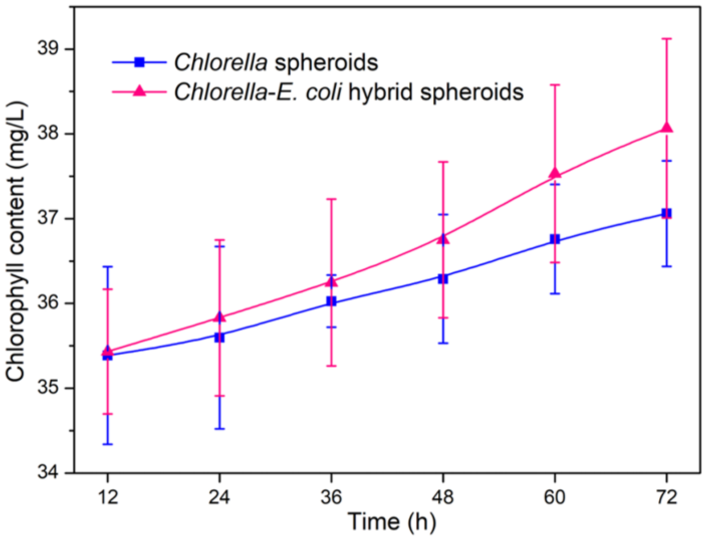

Figure S27. Plot of time-dependent changes in chlorophyll concentration in *Chlorella* cell-based spheroids (blue) and *Chlorella*/*E. coli* hybrid spheroids (pink). Error bars indicate standard deviations (n=3).

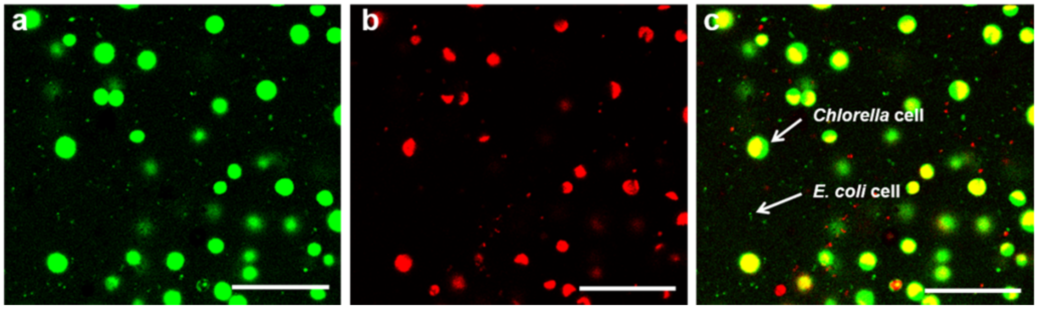

Figure S28. Cell viability after disassembly of *Chlorella*/*E. coli* hybrid spheroids. Green (a), red (b) and overlay (c) confocal fluorescence images of *Chlorella* and *E. coli* cells after the complete disassembly of *Chlorella*/*E. coli* micro-reactors at 108 h. Green fluorescence is from the hydrolysis of FDA of living cells. Red fluorescence is from intracellular chlorophyll (large objects, *Chlorella* cells) and dead bacterial cells via PI staining (small objects, *E. coli*), respectively. Scale bars, 25 µm.

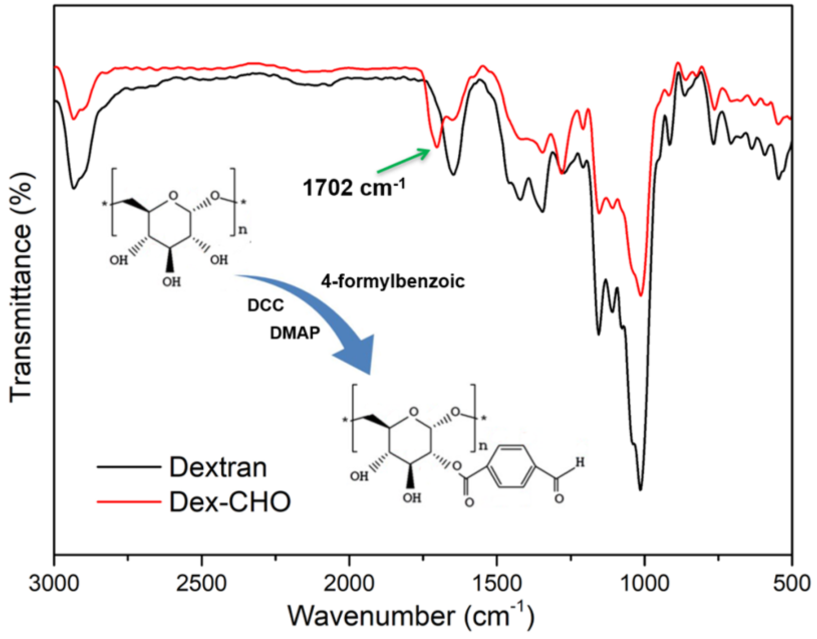

Figure S29. FTIR spectra of dextran (black line) and Dex-CHO (red line) confirming successful conjugation between -OH groups in dextran and carboxyl of formyl benzoic acid. The absorption peak is observed at 1702 cm^{-1} (Dex-CHO, formyl benzoic acid, C=O stretch).

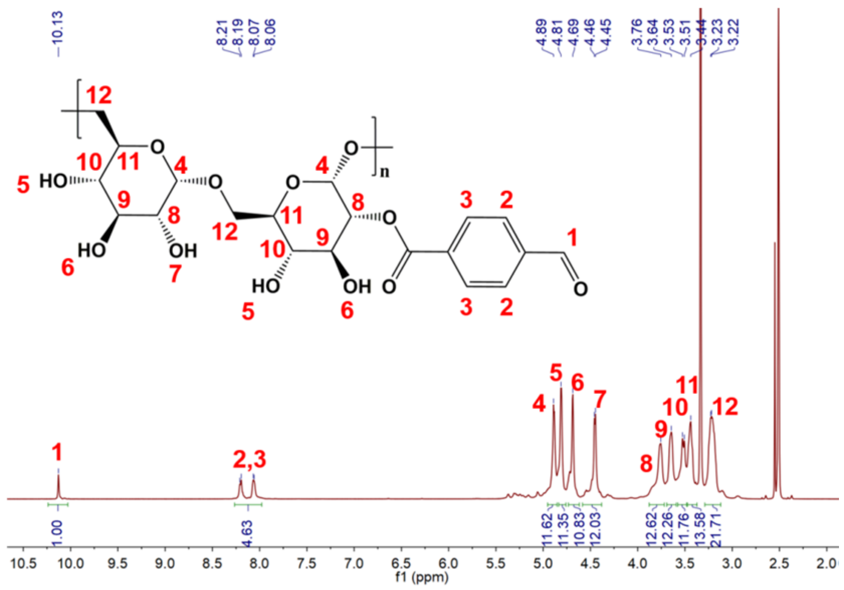

Figure S30. ^{1}H-NMR spectrum of Dex-CHO in $(CD_3)_2SO$. Based on the integrated intensity of the aldehyde resonance at 10.13 ppm, every 10 glucose units have one benzyl aldehyde group on average.

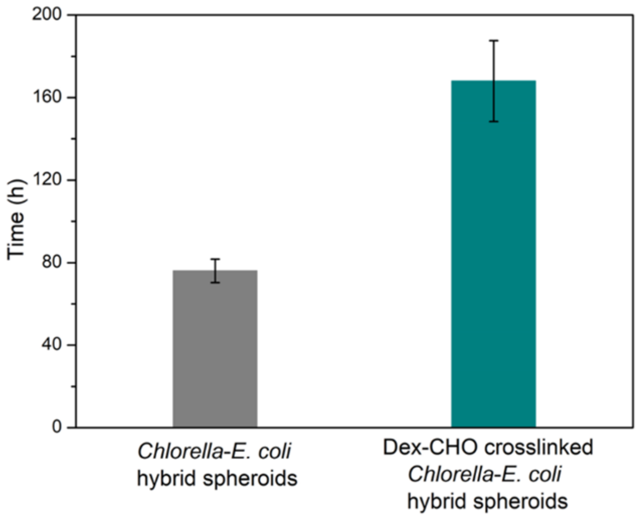

Figure S31. Hydrogen duration periods of non-crosslinked (left) and Dex-CHO crosslinked (right) *Chlorella*/*E. coli* hybrid spheroids. Error bars indicate standard deviations (n=3).

Responses to Reviewer #2:

The study exploited utilization of water-in-water dextran-in-PEG emulsion micro-droplets for encapsulating microalga *Chlorella pyrenoidosa* or assembling *Chlorella*/*E.coli* for the photosynthetic production of hydrogen under air. I am not convinced that the study shows enough novelty for publication in Nature Communications. I have the following major concerns, and I recommend rejection of the manuscript in its current form.

Response: We have addressed each of the Reviewer's comments (please see below). We disagree that the manuscript is not sufficiently novel for NCOMM. Indeed, reviewer 1 writes: *"The work demonstrates a clever approach to develop aqueous two-phase system-based microniches with dual functionality to enable photobiological production of hydrogen. Overall, this is a timely work that combines a unique multiphasic system with synergism between two types of biological cells to achieve a potentially useful function of hydrogen production."*

In addition, we now show that the duration of hydrogen production can be increased by further modifications of our procedures (please see response 5 above to comments from

Reviewer 1).

In brief, the advances in knowledge relate to:

(i) **Spatial organization of two different populations of living cells:** Recently, the emerging topic on living cell self-assembly is attracting much attention. However, the development of new techniques capable of orchestrating the assembly of living cells into multicellular ensembles with synergistic structure and function remains a considerable challenge. Our work exploits the aqueous two-phase separation of dextran-in-PEG emulsion micro-droplets and develops a new methodology for the capture, spatial organization and immobilization of algal cells or algal/bacterial cells, which then allow for the high throughput fabrication of photosynthetic microbial micro-reactors comprising spatially segregated communities of algal or algal/bacterial cells.

(ii) **Functionality switching between single cell and organized cell colony:** We demonstrate that cooperation between the respiration of *E. coli* and the physical barrier of the close-packed *Chlorella* cells allows a functionality switching of the single *Chlorella* cell colony from normal photosynthetic oxygen production to hydrogen production from the organized *Chlorella* cell colony. To the best of our knowledge, the demonstration of spatially dependent symbiosis in the constructed micro-niches of segregated communities of algal and non-photosynthetic bacterial cells has not been reported, especially with regard to their synergistic use to enhance hydrogen production.

Specific questions:

1. Developing stable water-in-water dextran-in-PEG emulsion micro-droplets is not anything new. As early as in 2016, Song et al. (Nature Communications, 7:12934, DOI: 10.1038/ncomms12934) developed similar technology, i.e., stable water-in-water dextran-in-PEG emulsion system, but this paper was not cited in the current manuscript.

Response: Many thanks for the suggestion. As stated in the reviewer's comment, the stabilization of all aqueous emulsions have been widely studied, and various efficient stabilizers such as inorganic nanoplates (*ACS Macro Lett.*, **2015,** 4, 965-968), rod-like cellulose nanocrystals (*ACS Macro Lett.*, **2016,** 5, 283-286), polydopamine particles (*Biomacromolecules*, **2019,** 20, 204-211), liposomes (*Nat. Commun.*, **2014,** 5:4670), or

protein particles with various shapes (*Langmuir*, **2013,** 29, 10658-10664; *Nat. Commun.*, **2016,** 7:12934; *Langmiur*, **2016,** 32, 7189-7197), have been employed to obtain stable water-in-water emulsions.

We apologize for not citing the paper mentioned by the reviewer (Nature Communications, 7:12934, DOI: 10.1038/ncomms12934) in the original manuscript. **The suggested paper is now cited in the main text as reference 9 (along with an additional Ref 10).**

2. The authors claim that a major contribution of the current study is the spatial organization and immobilization of algal or algal/bacterial cells which creates anaerobic niche in the core of the micro-droplets and induces production of hydrogen. While the basic concept has been demonstrated in a previous study (Ref 24), the current study does not show any groundbreaking progress in innovation. Utilizing w/w immobilization technique improves the stability of the assembly of cells but disassembly occurs over time and completely disassembled after 5 days (Figure S23).

Response: Although we agree that Tang et al (*Angew. Chem. Int. Ed.* **2015,** 54, 11961-11965) reported hydrogen production in air from algal cells housed within a mineralized core-shell structure, their method employed inorganic silicification rather than the living assembly of multiple cell colonies in dynamic w/w emulsion droplets. In our opinion, the experimental systems are fundamentally different as the novelty of our conceptual approach is based on the spatial organization and functional switching of multicellular microbial communities that can operate synergistically. As summarized on page 18: "*In conclusion, we developed a novel strategy for the preparation of populations of discrete microscale microbial reactors with spatial segregated multicellular micro-niches capable of aerobic or anaerobic photosynthesis at room temperature in air."* This is not a concept applicable to the immobilization of algal cells in silica.

We have also undertaken addition experiments to prolong the duration of hydrogen production (please see comments to point 5 from Reviewer 1). Whilst we have not yet achieved long-term continuous production, our opinion is that further developments will be possible and we have made some suggestions for further work in the revised Conclusions. But our principle focus is to demonstrate the proof-of-concept.

The following new text and references have been added to the Conclusions:

"Overall, our methodology provides a proof-of-principle for utilizing aqueous two-phase separated droplets as vectors for controlling algal cell organization and photosynthesis in synthetic micro-spaces. The procedure is facile and capable of high throughputs for modulating algal cell functionality towards hydrogen production without impairing the viability of the living cells. Moreover, it should be possible to combine our methodology with more complex bioengineering approaches involving sulfur deprivation,[24] genetically modified oxygen-tolerant [FeFe]-hydrogenases[25] or cellular surface modifications.[27] Compared with synthetic hydrogen producing systems,[30] the limited rates and yields in the multicellular spheroids remain challenging aspects of future work. In this regard, incorporating chemical-based hydrogen generating machinery[31, 32] or antennae-reduced mutants[33] into the algal cell spheroids could be promising strategies. More generally, our approach provides the possibility for modulating the functionality of other living cells; for example, the droplet-based microbial systems can be readily extended towards ethanol production via the programmed capture of large numbers of yeast cells within the multicellular spheroids (**Figure S32**)."

3. Additionally, the hydrogen production stopped after 72 h (Figure 5h), and the authors did not have appropriate discussion of possible reasons.

Response: As this question was also raised by Reviewer 1, we include below a repeat of our response.

We undertook further experiments to attempt to prolong hydrogen production by covalently fixing the formed multicellular ensembles to minimize cell proliferation and spheroid disassembly. To do so, aldehyde-functionalized dextran was specially synthesized and used as a cross-linker to further fix the formed multicellular spheroids in the dextran phase. As a consequence, hydrogen production in the optimal bioreactor system (*Chlorella*/*E. coli* hybrid spheroids) was successfully prolonged from 72 to 168 h.

Details of the new experiments have been now incorporated into the "Materials and Methods" section in the SI labelled. The following text has been added on page 2 and page 4/5 of the SI:

Page 2, SI: "… dextran (MW 70 kDa, Pharmacia), 4-Formylbenzoic acid (Energy Chemical, 98%), N,N-dicyclohexylcarbodiimide (DCC, Energy Chemical), 4-(dimethylamino) pyridine (DMAP, Sigma)"

Page 4/5, SI: "**Synthesis of aldehyde-functionalized dextran (Dex-CHO).** The functionalized dextran was prepared as follows. Mixtures of dextran (2 g, 0.0285 mmol, MW 70 kDa), 4-formylbenzoic acid (0.96 g, 6.4 mmol), and 4-(dimethylamino) pyridine (DMAP; 0.12 g, 0.984 mmol) were dissolved in 40 mL of dimethyl sulfoxide (DMSO), followed by the addition of N,N-dicyclohexylcarbodiimide (DCC; 1.2 g, 5.83 mmol). The system was stirred at room temperature for 18 h and then the impurity removed by filtration. The Dex-CHO product was obtained as a white solid after precipitation in a mixture of ethyl acetate and petroleum ether with a volume ratio of 1 : 9. The white solid was dissolved in the deionized water, and any insoluble impurities was removed by filtration, and then freeze-dried.

Construction of crosslinked robust *Chlorella*/*E. coli* multicellular spheroids. Algal/PEGylated bacteria cell mixtures (OD_{600} (*E. coli*) : OD_{750} (*Chlorella*) = 1 : 80, 120 µL) were added to 360 µL of an aqueous solution of dextran (120 µL, 160 mg/mL), denatured BSA particles (120 µL, 3 wt%) and Dex-CHO (120 µL, 2.4 wt%). The w/w emulsion droplets were slowly transferred into a hyperosmotic PEG solution dissolved in 10 mM PBS (pH = 7.4, 50 % w/w, MW 2000 Da) and left for 20 min to induce simultaneously shrinking of the droplets, compaction of the entrapped *Chlorella* and *E. coli* cells as well as the crosslinking reaction between Dex-CHO and the BSA particles. The vials were left unstirred and the sedimented spheroids collected by carefully removing the supernatant followed by washing several times using DI water."

The following revised texts have been added to the Abstract and on page 16 of the main manuscript:

Abstract: "…Moreover, the duration of hydrogen production is further prolonged by covalent crosslinking of the droplet microenvironment."

Page 16: "As the *Chlorella* and *Chlorella*/*E. coli* micro-reactors were stabilized only by non-covalent interactions along with *in situ* compaction and hydrogelation of the denatured BSA microparticles during hyperosmotic compression, the spheroids disassembled over time as the cell colonies proliferated (**Figure S25-S27**). As a consequence, hydrogen production was terminated typically within 72 h even though typical levels of cell viability after disassembly at 108 h were 68 and 96% for *E. coli* and *Chlorella* cells, respectively (**Figure S28**). To prolong the lifetime of the hybrid bioreactor, we included an aldehyde-functionalized dextran (Dex-CHO) into the w/w dextran-rich emulsion droplets to chemically crosslink the BSA protein hydrogel *in situ*. The resulting spheroids were physically more robust and displayed extended periods of hydrogen production over 168 h (**Figure S29-S31**)."

New data has been added; Figs S26, S27, S28, S29, S30, S31

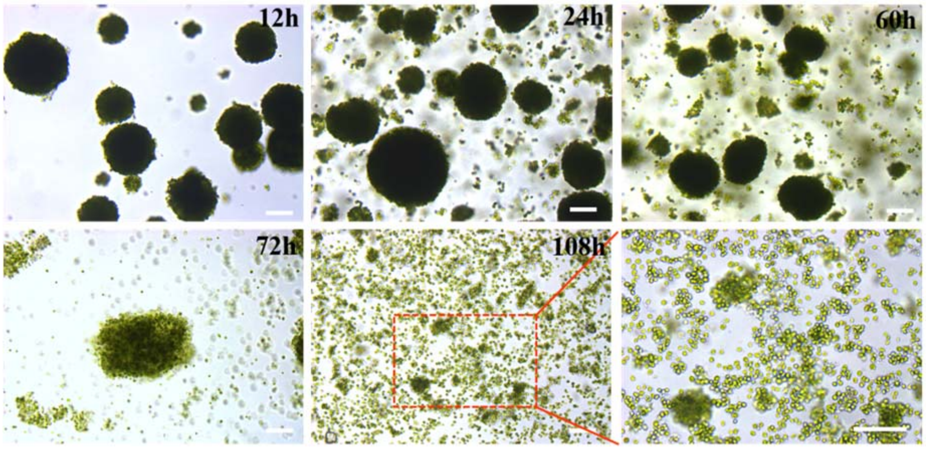

Figure S26. Optical microscopy images showing the gradual time-dependent disintegration of *Chlorella*/*E. coli* hybrid spheroids undergoing continuous photosynthesis in TAP culture medium. Scale bars, 50 µm.

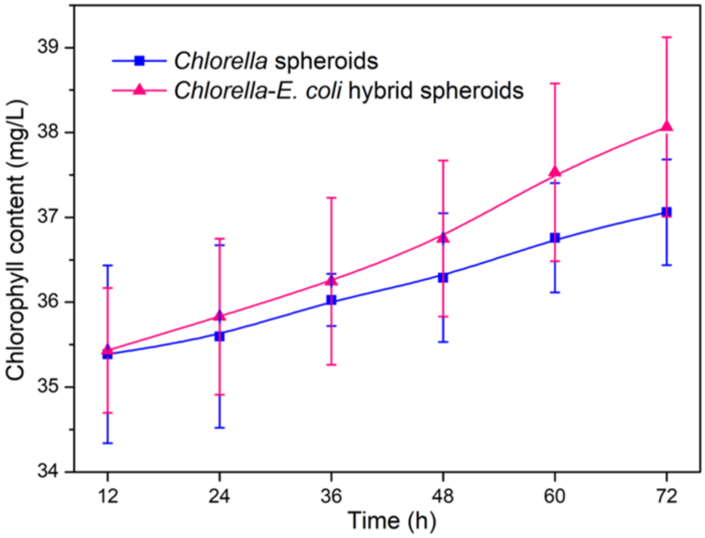

Figure S27. Plot of time-dependent changes in chlorophyll concentration in *Chlorella* cell-based spheroids (blue) and *Chlorella*/*E. coli* hybrid spheroids (pink). Error bars indicate standard deviations (n=3).

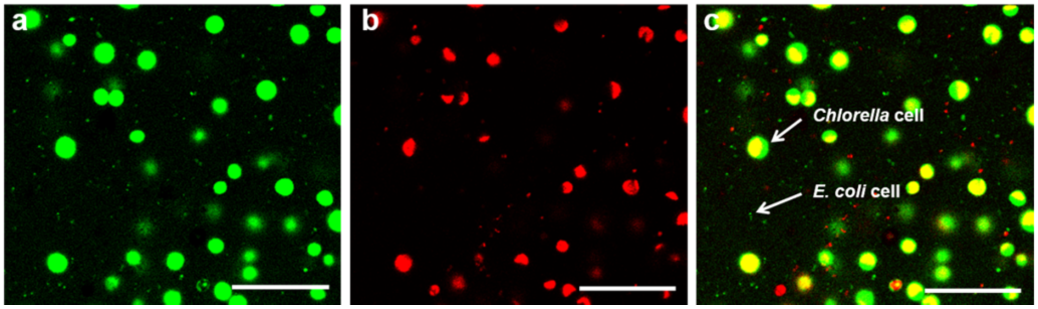

Figure S28. Cell viability after disassembly of *Chlorella*/*E. coli* hybrid spheroids. Green (a), red (b) and overlay (c) confocal fluorescence images of *Chlorella* and *E. coli* cells after the complete disassembly of *Chlorella*/*E. coli* micro-reactors at 108 h. Green fluorescence is from the hydrolysis of FDA of living cells. Red fluorescence is from intracellular chlorophyll (large objects, *Chlorella* cells) and dead bacterial cells via PI staining (small objects, *E. coli*), respectively. Scale bars, 25 µm.

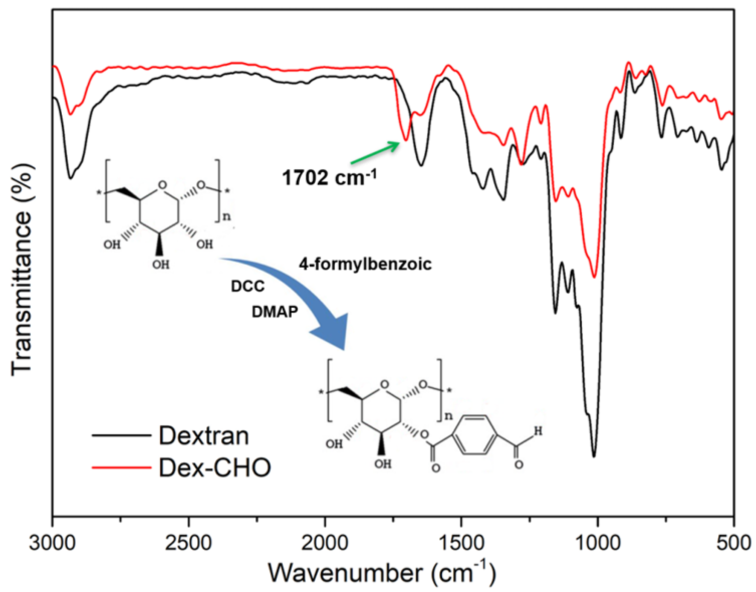

Figure S29. FTIR spectra of dextran (black line) and Dex-CHO (red line) confirming successful conjugation between -OH groups in dextran and carboxyl of formyl benzoic acid. The absorption peak is observed at 1702 cm^{-1} (Dex-CHO, formyl benzoic acid, C=O stretch).

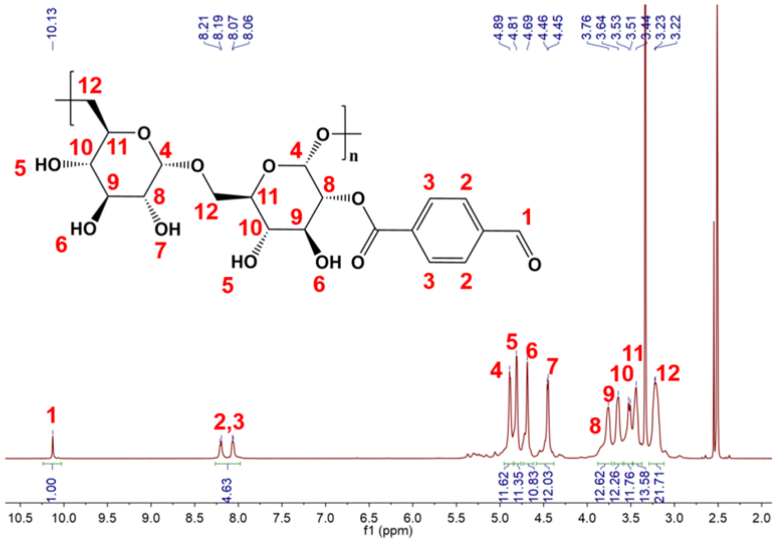

Figure S30. ^{1}H-NMR spectrum of Dex-CHO in $(CD_3)_2SO$. Based on the integrated intensity of the aldehyde resonance at 10.13 ppm, every 10 glucose units have one benzyl aldehyde group on average.

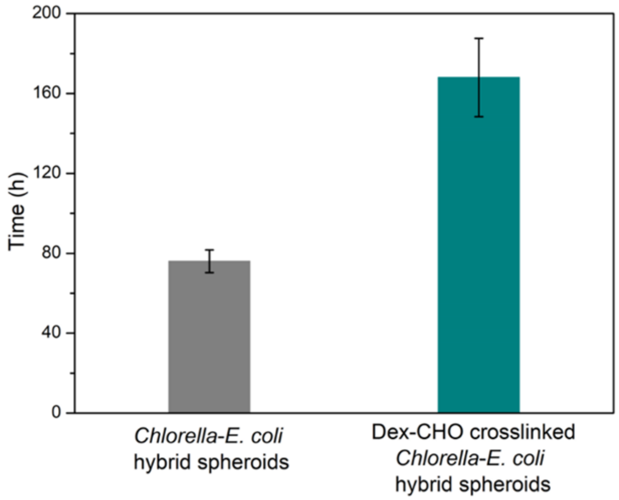

Figure S31. Hydrogen duration periods of non-crosslinked (left) and Dex-CHO crosslinked (right) *Chlorella*/*E. coli* hybrid spheroids. Error bars indicate standard deviations (n=3).

4. Page 11, first paragraph. It says the cells at the core were probably shield from the light source. However, hydrogen production consumes energy and there is no mentioning of where this energy comes from if without light/photosynthesis.

Response: We thank the reviewer for their thoughtful question. We agree that the energy used for hydrogen production must continue to come from the light source. Even though the *Chlorella* cells are tightly packed inside the formed spheroids, the peripheral cells do not shield all of the external light from reaching the core domain. Therefore, *Chlorella* cells located in the core still receive light illumination but at a relatively lower intensity, which then leads to the decrease of photosynthetic oxygen production. Thus, the anaerobic microenvironment inside the spheroids gradually emerges into enabling hydrogenase activation for hydrogen production.

To aid clarification, we have made the following revision in the main text on page 11: "We attributed the onset of an anaerobic micro-environment within the centre of the spheroids to partial shielding of the buried algal cells to the light source as well as restrictions in the diffusion of atmospheric oxygen into the core regions."

5. In Figure 5j, addition of DCMU inhibits PSII and also the hydrogen evolution, but the

authors did not explain why. How much DCMU was added into the system?

Response: DCMU is a type of photosynthetic inhibitor (*Proc. Natl. Acad. Sci.*, **2013,** 110, 7223-7228). Upon addition of DCMU, the electron transfer from Q_A to Q_B in PSⅡ is disrupted, thus terminating the hydrogen production of *Chlorella* spheroids or hybrid *Chlorella*/*E. coli* spheroids. We mention the generalities of this process in the main text on page 16, where we write "*In both cases, addition of 3-(3,4-dichlorophenyl)-1,1-dimethylurea (DCMU) inhibited hydrogen production (**Figure 5j**), confirming that anaerobic photosynthesis in the closely packed algal cells could be readily curtailed by disruption of the PSII electron transfer pathway*." As DCMU is often used in studies of photosynthesis, we do not feel that details of the intervention in the electron transfer chain are necessary. But we can add a reference if deemed necessary.

We have added the following experimental details in the Methods section of the SI (page 6): "**Hydrogen termination by DCMU.** The stock solutions of DCMU were prepared by dissolving 0.05 g of DCMU in 20 mL of acetone. For the termination of hydrogen production on the microbial reactors, 40 μL of DCMU solutions were added into the 2 mL of TAP culture medium with a final concentration of 200 μM."

6. In the section of "Synergistic hydrogen production in *Chlorella*/*E.coli* hybrid micro-reactors", the authors did not explain the *E. coli* cells respired on what substrate (bottom of page 14). Acetate in the TAP medium? Or excreted organic matters from the *Chlorella* cells?

Response: In nature, algae and bacteria are often inseparable and have sophisticated interactions with each other. Usually, algal cells provide dissolved/particulate organic matter such as carbohydrate, protein and amino acids for bacteria, while bacteria generate nutriments such as inorganic salts, vitamins and trace elements for the growth of algal cells (*Green Chem.*, **2014,** 16, 4716-4727; *Algal Res.*, **2017,** 28, 161-171). Accordingly, the substrate for the respiration of *E. coli* in the *Chlorella*/*E. coli* hybrid system is most likely associated with the excreta from the *Chlorella* cells.

We have added the following sentence on page 15 and two new references in the main text: "Moreover, as excreted algal-derived organic matter can be used as a substrate for bacterial respiration in natural and co-cultured algal/bacterial communities,[28, 29] we speculated that nutrient exchange between the co-located cells would lead to an enhancement in photobiological production."

7. Did *E. coli* cells grow and divide at all?

Response: Most of the *E. coli* were still alive in the presence of the intact *Chlorella*/*E. coli* multicellular spheroids. We used FDA and PI as the indicators to characterize the viability of the bacterial cells after disassembly of the *Chlorella*/*E. coli* spheroids. As shown in the **new Figure S28**, about 68 % of *E. coli* and 96 % of the *Chlorella* were alive after the complete disassembly of *Chlorella*/*E. coli* hybrid spheroids after 108 h.

These observations have been included in the revised manuscript and SI.

We have added the following text on page 16: "As a consequence, hydrogen production was terminated typically within 72 h even though typical levels of cell viability after disassembly at 108 h were 68 and 96% for *E. coli* and *Chlorella* cells, respectively (**Figure S28**)."

New Figure S28 (page 21, SI)

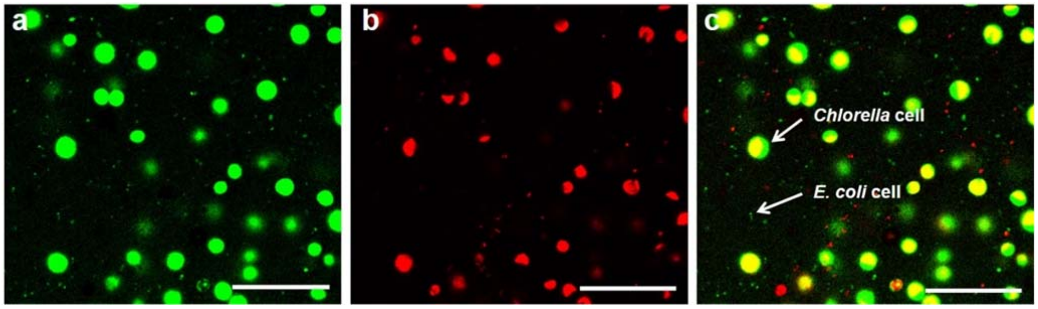

New Figure S28. Cell viability after disassembly of *Chlorella*/*E. coli* hybrid spheroids. Green (a), red (b) and overlay (c) confocal fluorescence images of *Chlorella* and *E. coli* cells after the complete disassembly of *Chlorella*/*E. coli* micro-reactors at 108 h. Green fluorescence is from the hydrolysis of FDA of living cells. Red fluorescence is from intracellular chlorophyll (large

objects, *Chlorella* cells) and dead bacterial cells via PI staining (small objects, *E. coli*), respectively. Scale bars, 25 µm.

8. In other words, the authors need to clarify what the energy flow is, what the shell cells do and what the cells at the core do and how they are connected. I feel a lot of detailed experiment-based analysis need to be done to answer these questions.

Response: The Reviewer raises a number of very interesting questions that go far beyond the scope of the current work, which is principally focused on*"..a methodology that offers a proof-of-principle for utilizing aqueous two-phase separated droplets as vectors for controlling algal cell organization and photosynthesis in synthetic micro-spaces and provides a step towards the bottom-up assembly of photobiological micro-reactors with multiple functionalities*." (page 3). The mechanistic details being considered by the Reviewer could take years to complete, and will be incorporated into aspects of our future work

Responses to Reviewer #3:

Overall this is a detailed and interesting study on a novel fabrication method using two-phase aqueous polymers to entrap algal cells (and other non-photosynthetic cells) into a relatively uniform (20-160 um) particles. Much of the paper especially the supplemental material addresses the methodological approaches such as shearing rpms, shrinking time, etc., Although these experiments do indicate the method is relatively well-controlled and although this is important, it seems this could have been published separately in a more methodological journal. In fact, I also think the paper would be complete and significant if it ended at Figure 4 and S19, thus omitting the E. coli cell inclusion work. Although quite well written and complete, there were also details that seem critical yet were not shown. For example, how denatured was the BSA from the heat treatment this could have been explored using CD, BN-PAGE, even tryptophan fluorescence. It was not even clear that the

BSA needed to be denatured?

Response: Many thanks for the reviewer's positive comments and the questions. As suggested by the reviewer, more details about the denatured BSA are now given in the revised manuscript.

The following text has been added on page 5: "Droplet coalescence was minimized by addition of specifically synthesized microparticles of denatured BSA (**Figure S1-S3**) to the dextran phase prior to emulsification (**Figure S4**)."

The following text related to the methods characterizing denatured BSA have been added to the SI "Methods" (page 2): "Circular dichroism (CD) spectral measurements were conducted using a Chirascan Plus CD spectrometer (Applied Photophysics Ltd, Leatherhead, UK). Samples were diluted with DI water to concentrations of 0.6 mg/mL in quartz cuvettes of 1 mm pathlength. Fluorescence spectroscopy was performed by a fluorescence spectrophotometer (PerkinElmer, USA, LS 55). Samples were diluted to a protein concentration of 1 mg/mL, and recorded at an excitation wavelength of 295 nm and slit widths of 6.0 nm (Excitation slit) and 3.0 nm (Emission slit)."

The following new SI figures have been added:

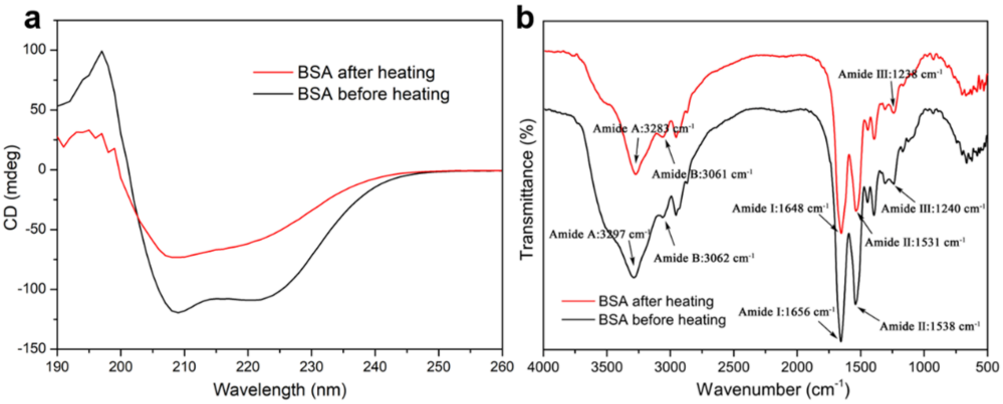

New Figure S2. Denaturation of BSA after heating. CD spectra (a) and FTIR spectra (b) of BSA before and after heating at 90 °C for 20 h. The CD results indicate that BSA before heating

contains 67.7% α-helix, 2.8% β-sheet and 7.0% β-turn, while BSA after heating contains 34.9% α-helix, 20.3% β-sheet and 10.0% β-turn, indicating heat-induced BSA unfolding. This is in agreement with the FTIR spectra of BSA before and after heat treatment, which show Amide A, Amide I and Amide III peaks with different levels of blue shifts. The blue shift of the Amide A band indicates breakage of hydrogen bonds on denaturation. Amide I band features are characteristic of α-helix and the shift to a lower wavenumber indicates a decrease in α-helix content. The Amide III band usually indicates β-sheet and β-turn conformations, and the shift to lower wavenumber demonstrates an increase in the content of β-sheet and β-turn.

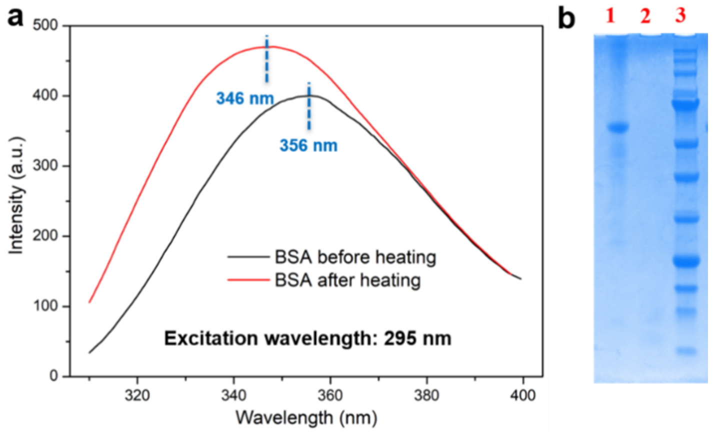

New Figure S3. Tryptophan fluorescence spectra (a) and SDS-PAGE profile (b) of BSA before and after heating at 90 °C for 20 h. Lane 1: native BSA; Lane 2: heated BSA; Lane 3: marker. From (a), the maximum emission wavelength of natural BSA is at 356 nm, while that of BSA heated at 90 °C for 20 h is blue shifted to 346 nm. This result demonstrates that tryptophan residues are blocked in the hydrophobic microenvironment because of denaturation and aggregation of BSA induced by the heat treatment. The aggregation is also confirmed by SDS-PAGE, in which compared to native BSA (Lane 1), heated BSA did not show any characteristic band (Lane 2), indicating the absence of monomeric BSA after heating treatment.

It was a bit disappointing to see that even after optimization, that hydrogen production stopped after ~72 hours and then the particles “dissolved” after five days. One would hope the “living” nature of the *Chlorella* cells would extend this activity longer than the in vitro PSI

hydrogen evolution yields shown by others such as the Golbeck lab and in reference 26. These researchers have demonstrated higher yield 5.5 mmol H_2/h/mg chlorophyll and a sustained activity for over 85 days? It would be very interesting to see the novel fabrication methods demonstrated here could be applied to include the incorporation of isolated PSI and a catalysis such as the platinum nanospheres or even a Ni/Fe hydrogenase. The integration of enzymatic oxygen scavenging methods (glucose oxidase and catalase) could also make this microsphere particle method compatible with more open environmental applications where anaerobiosis might not be feasible. Overall, it is an interesting and well done study, that may inspire other similar approaches in the field.

Response: We thank the reviewer's comments, and also really appreciate the highly aspiring suggestions and perspective, which we will definitely consider in future work. As the reviewer mentioned, in comparison with the artificial synthesized hydrogen-producing systems such as by the integration of the platinum nanospheres with isolated PSI or hydrogenase *etc*, our studies are not currently competitive for long-term hydrogen production. But developing mild and high throughput methods based on the spatial arrangement of living cells could have advantages especially for the modulation of functionality of single cell colonies and mixed cell communities.

We have undertaken additional experiments on extending the duration of hydrogen production from 72 to 168 h by *in situ* cross-linking of the hybrid spheroids. This new data is included in the revised manuscript (please refer to the response above to the 5th comment of the first reviewer).

Minor points/suggestions:

1. On Figure 2- it was unclear if the Red fluorescence on Panel 2K was the Chl autofluorescence or the fluorescence from the Nile Red from the denatured BSA?

Response: We have revised the caption of Figure 2k as follows: "Samples were prepared at a low cell number density (6.9×10^7 cells/mL) using non-labelled dextran and Nile Red-stained BSA particles. The captured algal cells display red and green fluorescence due to intracellular chlorophyll and cell-mediated release of fluorescein, respectively. The Nile Red-stained BSA particles are observed as a red fluorescence ring at the w/w droplet

interface.”

2. It is also surprising that the algal cells seem somewhat clumped together and only on the interior? Is this representative or simply one observation. More images would be preferred or possibly the use of flow cytometry to measure the number and distribution using the Slit-scan feature.

Response: In general, very few aggregates were observed when relatively low numbers of cells were encapsulated (1.2×10^8 cells/mL, **Figure 2f,g**), while clumping occurred at higher loadings (3.3×10^9 cells/mL, **Figure 2h,i**). The images in **Figure 2j-m** were recorded at very low cell number density (6.9×10^7 cells/mL) so that individual cells could be visualized.

3. With the low cell density ($6.9 \text{ x } 10^7$) there are only a few cells per droplet (I would guess ~20, yet this should be known with statistics using either LSCM or FACS). However, when you make the droplets using high cell density ($3.3 \text{ x } 10^9$) the number of cells goes up to 9000. This is a very large increase 450 times more yet only a 50-fold increase in cell density? This suggests some type of aggregation or algal clustering?

Response: It was difficult to count accurately the cell numbers per droplet at medium and high loadings using LSCM because of aggregation within the interior. However, the loading efficiency of the algal cells was close to 1.0 (100%) (minimal numbers of cells were observed in the continuous phase); we therefore calculated the statistical average cell number per droplet (n) using the following equation:

$$n = \frac{qcV_1}{\frac{V_2}{(4/3)\pi r^3}}$$

Where q was the encapsulation efficiency (1.0); c and V_1 were the cell density (cells/mL) and volume (mL) of used algal cells, respectively; V_2 was the volume of dextran phase (mL); r indicated the average radius of *Chlorella*-entrapped droplets (μm). The calculation gives n values of *ca*. 8700 and 180 for *Chlorella*-entrapped droplets prepared with cell densities of $3.3 \text{ x } 10^9$ or $6.9 \text{ x } 10^7$ cells/mL. Thus, the cell density was proportional to cell number per droplet, and appeared independent of preferential cell aggregation or clustering in the micro

dextran phase.

We have included the above equation and following text on page 4 of the SI "Methods" section: "The statistical average cell number per droplet (*n*) was calculated using the following equation:

$$n = \frac{\dfrac{qcV_1}{V_2}}{(4/3)\pi r^3}$$

where *q* was the encapsulation efficiency (*ca*. 1.0); *c* and V_1 were the cell density (cells/mL) and volume (mL) of used algal cells, respectively; V_2 was the volume of dextran phase (mL); and *r* the average radius of the *Chlorella*-entrapped droplets (μm). The calculation gave *n* values of *ca*. 8700 and 180 for *Chlorella*-entrapped droplets prepared with cell densities of 3.3 x 10^9 or 6.9 x 10^7 cells/mL, respectively."

4. Although this has been somewhat systematically studied in the Fig. S3-S9, there are still somewhat inconsistent results. For example, Fig. 3 with Fig. S7. The hyperosmotic shrinking seems to be very different. This would be interesting to explore more systematically.

Response: Many thanks for reviewer's careful reading and pointing this out. The original **Figure S7** was a control experiment related to **Figure 3**. It was our negligence that there were some mistakes in the caption of **old Figure S7**. We have now revised the original description "Volume contraction of *Chlorella*-loaded w/w emulsion droplets after immersion in hyperosmotic PEG solution" to "Volume contraction of w/w emulsion droplets after immersion in hyperosmotic PEG solution" **The legend has now been corrected (now Figure S10).**

5. Assuming diameter of 171 um before hyperosmotic shrinking (~1,964,000 um^3) but after the diameter has only reduced to 162 with a volume of 1,764,000 um^3. This is only a 11% reduction not the 50% shown in Fig. 3b.

Response: As **Figure 3b** refers specifically to the single droplet shown in **Figure 3a**, to avoid any misunderstanding, we have added the statistical diameter changes observed during

hyperosmotic shrinking as a **new Figure 3c**.

The new Figure 3c and caption (page 10) are as follows:

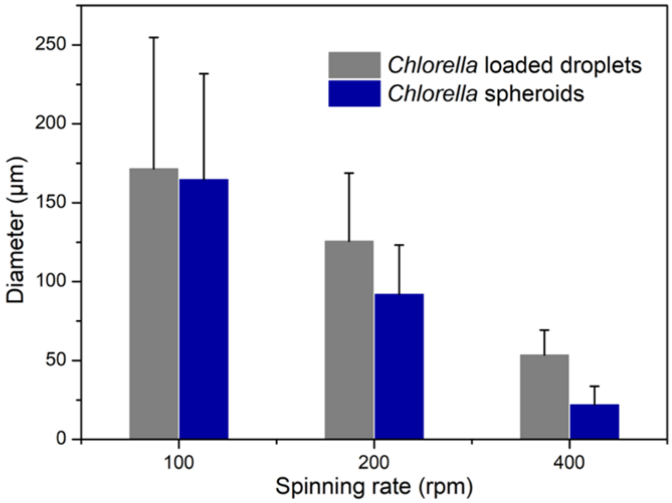

New Figure 3c. Plots of diameters of *Chlorella*-loaded droplets prepared at different spinning rates, before (navy blue) and after (deep cyan) hyperosmotic compression.

6. Figures 3E and F seem redundant to those images shown in Figure 2 K and I.

Response: Figure 3e,f is shown to confirm that the hyperosmotic compression process did not impose a negative influence on cell viability. This is different from the data in **Figure 2k,l** that shows the distribution of viable algal cells in the dextran droplets.

We have included the following text in the caption of Figure 3e,f (page 10) to clarify these differences. "(**e,f**) Confocal microscopy fluorescence images of *Chlorella* cell-based spheroids after FDA staining showing viable algal cells after hyperosmotic compression within the spheroids (e); red fluorescence is from intracellular chlorophyll (**f**); scale bars, 75 µm."

7. Explanation of large variation of size of their droplets? Is there an issue with reproducibility/controllability of the fabrication?

Response: The mean size of the water-in-water droplets was well controlled (from 180 to 20 µm) by varying the shear force (from 100 to 1000 rpm) such that the procedure showed

good reproducibility in this regard. However, the polydispersity was relatively high, which was expected due to the conventional emulsification techniques employed. But our approach allows for a simple, fast and large scale preparation of the droplets. Alternatively, microfluidic fabrication could be used to decrease the polydispersity.

8. With the photosynthetic *Chlorella* cells in the interior of these micro-reactors, are there any shading effects that are noted, or heterogeneity in productivity going deeper into these droplets?

Response: Many thanks for the insightful question. We have not investigated whether there are distinct gradients in hydrogen production due to shading effects.

9. I was hoping to get more of a broad context in the conclusion to get an idea of where to place this study in comparison to previous work on biomass production by whole cells.

Response: The following text and references have now been added to the Conclusions section of the revised manuscript (page 19). "Overall, our methodology provides a proof-of-principle for utilizing aqueous two-phase separated droplets as vectors for controlling algal cell organization and photosynthesis in synthetic micro-spaces. The procedure is facile and capable of high throughputs for modulating algal cell functionality towards hydrogen production without impairing the viability of the living cells. Moreover, it should be possible to combine our methodology with more complex bioengineering approaches involving sulfur deprivation,[24] genetically modified oxygen-tolerant [FeFe]-hydrogenases[25] or cellular surface modifications.[27] Compared with synthetic hydrogen producing systems,[30] the limited rates and yields in the multicellular spheroids remain challenging aspects of future work. In this regard, incorporating chemical-based hydrogen generating machinery[31, 32] or antennae-reduced mutants[33] into the algal cell spheroids could be promising strategies. More generally, our approach provides the possibility for modulating the functionality of other living cells; for example, the droplet-based microbial systems can be readily extended towards ethanol production via the programmed capture of large numbers of yeast cells within the multicellular spheroids (**Figure S32**)."

10. In trying to explain the lack of hydrogen production in the 22 um sized spheroids as compared to the 92 um, the authors claim that this is due to "to the increased number of algal cells in the aerobic surface regions". Since the particle size is smaller and the surface area much lower, I think the authors actually mean an increased percentage of Algal cells are in an aerobic environment and/or there is a drastic loss of cells in the anaerobic environment. The number of cells is certainly overall less (not more as stated) in the 22 um vs the 92 um.

Response: We agree with the Reviewer and have revised the sentence on page 12 as follows: "… in agreement with the relatively high oxygen production under these conditions due to the increased percentage of algal cells in the aerobic surface regions (**Figure 4a**)."

11. On page 12, It is also interesting that the hydrogen yield decreased when they go from 92-165 um, this may be due to the shading as suggested yet this might be a key area for further optimization. It might also be interesting to incorporate antennae reduced mutants to see if increasing the anaerobic environment while also increase light penetrance could further boost the hydrogen yield.

Response: Many thanks for these interesting ideas.

We have added these points with references in the Conclusions (see revised text shown in response 9 above).

Reviewers' comments:
Reviewer #1 (Remarks to the Author):

The authors have addressed the comments and questions satisfactorily.

Reviewer #2 (Remarks to the Author):

After re-evaluating the revised manuscript, I feel there is some novelty as a proof-of-principle study for utilizing dextran-in-PEG emulsion micro-droplets to spatially organize separate populations of living cells. Although the micro-droplets still have durability issues – ensembles only sustaining a few days, the fabrication method demonstrated in this study is sound. However, the biology part is a bit weak and is not thoroughly interpreted and discussed.

Biosynthesis of hydrogen requires orchestration of the energy input and oxygen scavenging. According to the presumption depicted in the legend of Figure 1, the "shell domain" conducts oxygenic photosynthesis and the "core domain" conducts oxygenic photosynthesis and H2 biosynthesis. There is a lack of illustration (in the figure or figure legend) & explain (in the text) of the fate of O2, which is crucial for the functionality of O2-sensitive hydrogenases. Based on the basic mechanism of oxygenic photosynthesis (powered by PSII), whenever 2 electrons (which is required for producing 1 H2) are produced through splitting 1 water molecule at the PSII complex center, half O2 is released at the same time. In order to scavenge this half O2, 2 electrons are consumed. That means, using light as the sole energy source without input of any other types of reducing agent, the net yield of free electrons (which could be used for biosynthesis of H2 by H2ase) is 0, under non-oxygenic
(and thereby H2-producing) conditions. The net H2 yield is therefore 0. In other words, H2 biosynthesis can not be simply based on PSII-powered oxygenic photosynthesis.

Alternatively, algal/bacterial cells could respire on storage compounds, such as saccharides and lipids, to scavenge O2, and therefore free up some electrons for H2 generation, which is probably the case in this study. In particular, in Figure 4a, the O2 level in the case of 92 um micro-droplets dropped along the time course, suggesting faster O2 scavenging than generation, which implies reducing storage compounds were consumed during the process. The energy source for H2 biosynthesis in the current experimental setting in this study boils down to be dependent on storage compounds, not photosynthesis through PSII. Addition of DCMU not only blocks the electron transport chain in photosynthesis but also inhibits O2 scavenging by respiration (Canadian Journal of Botany, 1972, 50(1): 13-21, https://doi.org/10.1139/b72-003). Therefore, Figure 5j does not prove H2 biosynthesis is directly associated with the electron flow originating from PSII. Given the H2 biosynthesis depends on
storage compounds, it may also help to explain why H2 production diminished after a few days.

"anaerobic photosynthesis" sometimes is also called anoxygenic photosynthesis, means photosynthesis that does not involve generation of O2 at all. Using "anaerobic photosynthesis" to indicate PSII associated oxygenic photosynthesis is misleading. I suggest

the authors consider replacing the term, “anaerobic photosynthesis”, which has been used many times in the manuscript.
Overall, the authors need to clarify what the energy flow is, what the shell cells do and what the cells at the core do and how they are connected and how the process is orchestrated.

Reviewer #3 (Remarks to the Author):

This manuscript NCOMMS-20-07268A on “Photosynthetic hydrogen production by droplet-based microbial micro-reactors under aerobic conditions" has been revised and substantially expanded. I have read the response to all three reviewers. I am impressed with the substantial amount of new data provided: a new Figure 5H and new Supp. Figures 8, 26-31. There was a substantial response to each reviewer and most, but not all issues have been improved. The response to reviewers is quite long (31 pages), yet a major section of the response to Reviewer 1 was directly repeated for Reviewer 2. This accounts for ~6 pages of response. The addition of Supp. Figure 28 was actually discussed 3 times.

Remaining concerns:

All three reviewers were disappointed to see the loss of hydrogen evolution after only 72 hours, especially since in vitro methods are much longer lasting. Although this loss of activity has still not been investigated in detail , they suspect that this may be due so some particle instability. The authors have modified their fabrication method to include a Dex-CHO crosslinking. This extended the duration of hydrogen evolution out to ~160 hrs. Although this is shown in Figure S31, I would really like to see this data represented in the same format as Fig. 5H. This would then be the moved to the main body of the paper as probably the last figure. Although this work is touted as a proof-of-principle, this new data gives one direction that may be used to improve the performance.

Summary- I feel the paper is now improved, and most issues are now resolved. It is still quite long yet I feel it can be accepted with only very minor changes: moving Figure S31 up into the main body.

Responses to Reviewer #1:

Response: Many thanks for the reviewer's acceptance of our work.

Responses to Reviewer #2:

Response: We thank the referee for their time on reviewing our work, and very much appreciate their insightful comments on the biological mechanisms of hydrogen production, which help to improve the paper. We agree with the comments and have incorporated them into the manuscript as follows.

1/ Figure 1c legend (page 4) has been revised to take into account the related mechanism:

"(**c**) Cell-mediated depletion of oxygen over time in the hydrogel matrix (yellow triangular network) generates hypoxic (interior, cyan) and aerobic (surface, green) micro-niches due to light shading of the algal cells in the core domain by the outer shell of *Chlorella* cells. Depending on the size of the spheroids, photosynthetic oxygen generation is decreased in the core such that respiration becomes dominant over photosynthesis resulting in the net depletion of cellular storage compounds, hypoxic conditions, hydrogenase activity and hydrogen production in daylight under air. Corresponding reactions: Shell domain: $H_2O \rightarrow ½O_2 + 2H^+ + 2e^-$ (PSII). Core domain: $H_2O \rightarrow ½O_2 + 2H^+ + 2e^-$ (PSII) and $2H^+ + 2e \rightarrow H_2$ (hydrogenase)."

2/ The following supplementary discussions have been added to Page 19 of the main text:

"Significantly, the *E. coli*-enriched outer shell not only acts as a shield of the light source but also consumes oxygen produced from algal cell photosynthesis in the core of the spheroids, both of which facilitate H_2 biosynthesis provided that the cellular storage compounds are not depleted and that the spheroids do not disassemble due to continued cell proliferation. In the latter case, crosslinking of the hydrogel matrix within the multicellular spheroids resulted in an extension in continuous hydrogen production to 168 h."

3/ The following text have been added on Page 11 to demonstrate the fate of oxygen from shell domain:

"However, increasing the mean size of the light-exposed spheroids from 22 to 92 µm resulted in a decrease in the dissolved oxygen concentration from 4.04 to 0.25 mg/L over *ca.* 12 h (**Figure 4a**) and time-dependent decreases of the intracellular ATP concentrations (**Figure 4b**). This suggested that as the spheroid core became larger, respiration became dominant over photosynthesis such that there was a net consumption of oxygen molecules. We attributed this transformation to the onset of a hypoxic micro-environment within the centre of the spheroids due to partial shielding of the buried algal cells to the light source as well as restrictions in the diffusion of atmospheric oxygen into the core regions. As a consequence, *Chlorella* cells within each micro-reactor became segregated into hypoxic or aerobic micro-niches depending on whether they were located in the core or shell of the spheroid, respectively."

4/ **We have replaced the term "anaerobic photosynthesis" with "hypoxic photosynthesis" throughout the manuscript to avoid misleading the readers.**

5/ To clarify the function of DCMU in terminating hydrogen production, the following revised text have been added to the **Page 16 of the main text**:

"In both cases, addition of 3-(3,4-dichlorophenyl)-1,1-dimethylurea (DCMU) inhibited hydrogen production (Figure 5j). This was consistent with the simultaneous DCMU-mediated disruption of the PSII pathway and inhibition of respiration, and implied that both electron transfer and oxygen-scavenging were important in determining hydrogen production."

Responses to Reviewer #3:

Response: Many thanks for the reviewer's acceptance of our work. As suggested**, we have incorporated Figure S31 into Figure 5h in the main text Page 17.**

Reviewers’ comments:
Reviewer #2 (Remarks to the Author):

The authors have addressed all my questions in the revised manuscript.

Responses to Reviewer #2:

Response: Many thanks for the reviewer's acceptance of our work.
